# Protein X of Hepatitis B Virus: Origin and Structure Similarity with the Central Domain of DNA Glycosylase

**DOI:** 10.1371/journal.pone.0023392

**Published:** 2011-08-05

**Authors:** Formijn J. van Hemert, Maarten A. A. van de Klundert, Vladimir V. Lukashov, Neeltje A. Kootstra, Ben Berkhout, Hans L. Zaaijer

**Affiliations:** 1 Laboratory of Experimental Virology, Department of Medical Microbiology, Center for Infection and Immunity Amsterdam (CINIMA), Academic Medical Center, University of Amsterdam, Amsterdam, The Netherlands; 2 Laboratory Clinical Virology, Department of Medical Microbiology, Center for Infection and Immunity Amsterdam (CINIMA), Academic Medical Center, University of Amsterdam, Amsterdam, The Netherlands; 3 Laboratory of Experimental Immunology, Department of Medical Microbiology, Center for Infection and Immunity Amsterdam (CINIMA), Academic Medical Center, University of Amsterdam, Amsterdam, The Netherlands; Institut Pasteur, France

## Abstract

Orthohepadnavirus (mammalian hosts) and avihepadnavirus (avian hosts) constitute the family of Hepadnaviridae and differ by their capability and inability for expression of protein X, respectively. Origin and functions of X are unclear. The evolutionary analysis at issue of X indicates that present strains of orthohepadnavirus started to diverge about 25,000 years ago, simultaneously with the onset of avihepadnavirus diversification. These evolutionary events were preceded by a much longer period during which orthohepadnavirus developed a functional protein X while avihepadnavirus evolved without X. An *in silico* generated 3D-model of orthohepadnaviral X protein displayed considerable similarity to the tertiary structure of DNA glycosylases (key enzymes of base excision DNA repair pathways). Similarity is confined to the central domain of MUG proteins with the typical DNA-binding facilities but without the capability of DNA glycosylase enzymatic activity. The hypothetical translation product of a vestigial X reading frame in the genome of duck hepadnavirus could also been folded into a DNA glycosylase-like 3D-structure. In conclusion, the most recent common ancestor of ortho- and avihepadnavirus carried an X sequence with orthology to the central domain of DNA glycosylase.

## Introduction

The hepatitis B virus (HBV) particle contains a partially double stranded DNA genome of about 3200 base pairs [1], [2] with four partially overlapping reading frames encoding the C, S and X proteins and the error-prone viral reverse transcriptase or polymerase [3], [4]. Besides human HBV, the subfamily of Orthohepadnaviridae includes similar virus strains isolated from gorilla, orangutan, chimpanzee, gibbon, woolly monkey, chuck and squirrel species. Infections by Avihepadnaviridae have been demonstrated in duck, goose, heron and stork species. About 300 million patients worldwide carry a chronic HBV infection [5], which causes the death of over one million persons annually by liver failure or hepatocellular carcinoma [6]. Transgenic mice expressing the X protein in liver were prone to develop hepatocellular carcinoma [7]. However, the cellular pathways along which X induces hepatocellular carcinoma are fragmentary documented [8]. The X protein is 154 amino acids in size and displays direct or indirect interaction with host factors, thus modulating a plethora of cellular processes. Protein X interferes with transcription, signal transduction, cell cycle progress, protein degradation, apoptosis and chromosomal stability [9]–[11]. More specifically, heterodimer complex formation of X with its cellular target protein (HBX interacting protein, HBXIP) has been demonstrated, triggering deregulation of centrosome dynamics and mitotic spindle formation [12]. Another interaction involves DDB1 (Damaged DNA Binding Protein1), in which case protein X redirects the ubiquitin ligase activity of CUL4-DDB1 E3 complexes, which are intimately involved in the intracellular regulation of DNA replication and repair, transcription and signal transduction [13]. The X protein is well conserved among (mammalian) orthohepadnavirus, but absent in avihepadnavirus. Similarity of X with host cellular proteins appeared to be below or near the threshold level of detection and a crystal model of its 3D-structure is currently not available.

Here, we present an *in silico* generated model of the X tertiary structure. The X model of choice is the best of the 5 alternative structures constructed by the modeling software. In docking experiments of the X structures with HBXIP or DDB1 into heterodimers, X model 1 outperforms most other models regarding the interface stability of these complexes. Amino acid residues in X proven to be critical for dimer formation with HBXIP are among the contact residues of the interface. In heterodimers of DDB1 with full-length protein X, the interfaces contain most of the H-box α-helical X residues that were described to be involved in a DDB1/H-box oligopeptide complex [13]. We have queried the PDB database for proteins displaying similarity with the X 3D-structure and found a striking similarity of X with members of the *MUG* family of DNA glycosylases, which are the key enzymes of the BER (base excision repair) pathway [14]. Even the hypothetical translation product of a vestigial X reading frame in duck hepadnavirus - after restoration of stopcodons into coding triplets [15] - showed a 3D-structure with significant similarity to *MUG* proteins. Protein-DNA docking experiments indicated a binding capability of X protein to an oligodeoxynucleotide that has been analyzed by X-ray in complex with E. coli *MUG* DNA glycosylase [16], [17]. From the evolutionary point of view, orthohepadnavirus and avihepadnavirus share a common protein X ancestor with orthology to DNA glycosylase.

## Materials and Methods

The NCBI reference set served as a source of human HBV sequences. Hepadnavirus sequences of non-human hosts (other primates, woolly monkey, chuck, squirrel and birds) were downloaded from GenBank. X protein sequences were derived by translation of the appropriate reading frame in complete viral genomes. An ancestral and a consensus sequence of human HBV X protein was constructed from the same collection by means of the ANCESCON server [18] and by BioEdit [19], respectively. An HBV genotype D consensus sequence was available [20]. Sequences with relatively high similarity were aligned by ClustalW [21] or by PROBCONS [22] in case of low similarity. Alignments were combined by the profile-to-profile option of MUSCLE [23] and subjected to rounds of manual refinement particularly at gap borders. The BEAST suite consisting of the modules Beauti, Beast, Logcombiner, TreeAnnotator v1.4.8, Tracer v1.4.1 and FigTree v1.2.3 [24] was used locally and distantly on the BEAST server of the Computational Biology Service Unit at Cornell University (http://cbsuapps.tc.cornell.edu/beast.aspx) for phylogenetic reconstruction and tMRCA (time of the most recent common ancestor) estimation. An uncorrelated lognormal relaxed molecular clock was assumed to act via the JTT amino acid replacement model [25] with gamma and invariant site heterogeneity. A constant population size was chosen as a demographic model. Identical analyses were done in parallel over a sufficient period of time to achieve convergence of Monte Carlo Markov chains. Relevant XML files are added as Supporting Information Files. DNA glycosylases are indicated by their PDB entry (lower case) with the chain identifier (upper case). The actual GenBank accession numbers are mentioned in table and figures. TimeTree [26] estimates of species divergence were used to calibrate BEAST analyses for the determination of the rate of amino acid replacements in animal DNA glycosylases. 3D-models were generated by submission to the *i*-TASSER server [27] and visualized by means of RasTop 2.2 (http://sourceforge.net/projects/rastop). Application of parent or template structures (server-detected or custom-supplied) is mentioned in the text. Protein-protein docking was achieved by ClusPro 1.0 [28] and by ClusPro 2.0, presently available as PIPER [29]. The usage of ALASCAN [30] for the identification of protein-protein interfaces and the calculation of interface stability has been described previously [31]. Distances between amino acid residues were estimated by means of Chimera v1.6 [32]. Protein-DNA docking was performed by means of PatchDock [33] and HEX [34]. Protein-DNA interfaces were analyzed by means of ProTorP [35]. The servers DALILITE [36] and MATRAS [37] were used for the detection and comparison of proteins with similarity to the tertiary structure of X. These servers employ different algorithms to solve identical queries. MATRAS offered the option to generate pairwise similarity matrices reflecting relative positions of ^β^C-atoms in polypeptide chains. Dendrograms were constructed by feeding these matrices into the neighbor-joining tree building facility of MEGA v4 [38].

## Results

### Mutational rates and evolutionary dates

**Figure 1:**
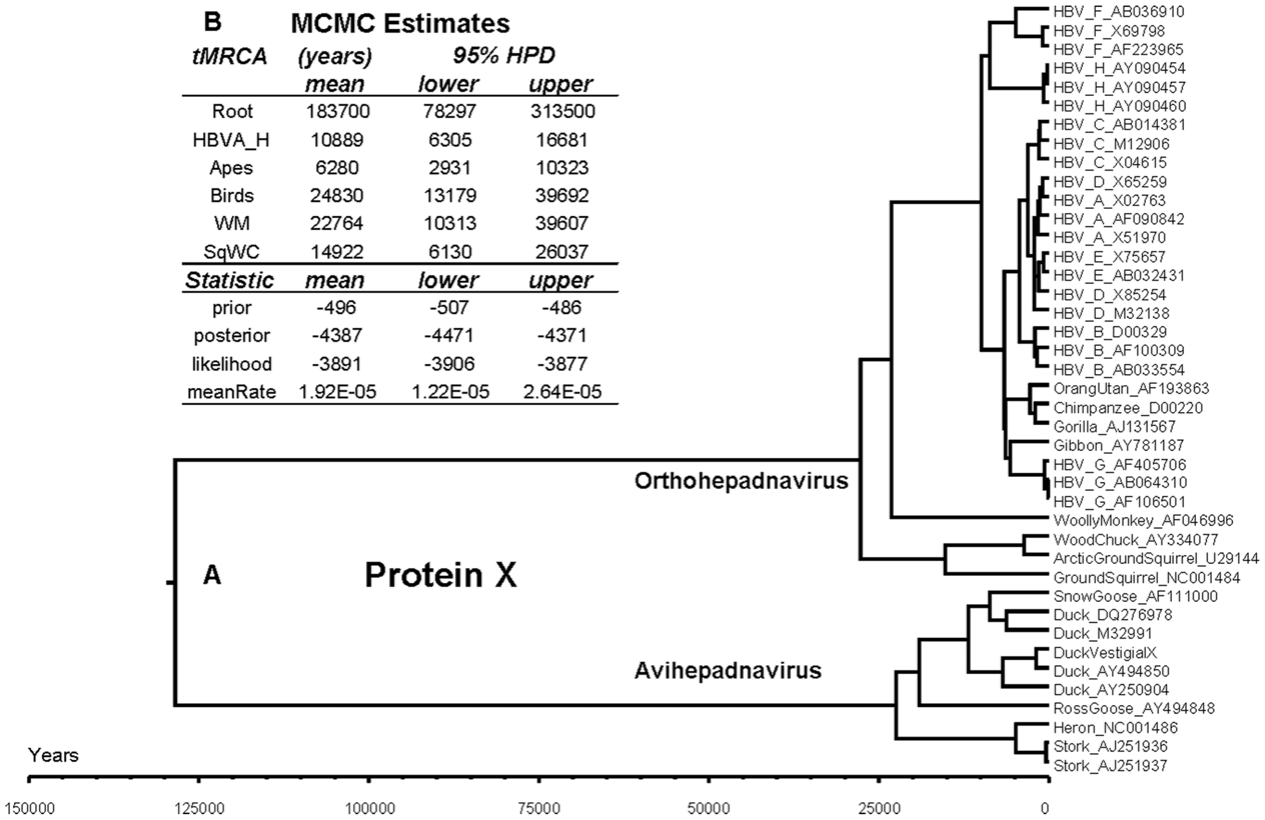
Phylogeny and divergence time estimates of hepadnaviral protein X. (A) Virus strains are indicated by the common name of their hosts and the GenBank accession identifier. The evolutionary sequence of events is displayed in tree format. In the sequence marked “DuckVestigialX”, stopcodons were replaced by coding triplets. In the other avian sequences, gaps were introduced at stopcodon sites in the vestigial X reading frame prior to translation into protein. (B) Monte Carlo Markov Chain (MCMC) estimates and parameter statistics are given without decimal numbers for mean values and highest posterior density interval (HPD). Minor differences between corresponding numbers in A and B are due to the stochastic character of the MCMC algorithm. The XML file used for BEAST analysis is provided as File S1.

A longitudinal study is available spanning the molecular evolution of hepatitis B virus over 25 years [39]. A rate of nonsynonymous mutations of about 2×10^−5^ amino acid replacements per site per year (r/s/y) was calculated from these data. This figure was used as a prior value for the fixed mean rate of amino acid replacement in protein X of the available hepadnavirus species. In birds infected with avihepadnavirus, X protein is not expressed. However, Lin & Anderson [15] have reported the presence of a vestigial X open reading frame in DNA of duck hepadnavirus. They deliberately reconstructed five stopcodons into coding triplets yielding a hypothetical translation product (138 AAs) with a hydrophilicity profile that closely matched that of mammalian X protein. Apparently, the nearly complete overlap of the vestigial X reading frame with functional polymerase and capsid coding sequences prevented the introduction of more deleterious frame shifts or deletions/insertions. In the Duck_VestigialX sequence, we replaced stopcodon positions by amino acid residues accordingly and modified the other avian X sequences with a gap at each stopcodon position. The results of Bayesian phylogenetics based on protein X show that divergence of orthohepadnavirus and avihepadnavirus occurred more than 125,000 years ago (Fig. 1, 95% HPD interval 78,297–313,500). Evolutionary events leading to the presently circulating virus strains started about 25,000 years ago for both orthohepadnavirus and avihepadnavirus (95% HPD interval 13,179–39,692). The clades of HBV in mammalian hosts correspond closely with the genotypes A-H. Protein X of HBV genotype G was found among the virus strains in apes instead of humans. Values for tMRCA emergence estimate the onset of divergence among the mammalian population at about 10,000 years ago (95% HPD interval 6,305–16,681). The corresponding BEAST xml file is provided as File S1. A tMRCA value of about 7,000 years ago (95% HPD interval 5,287–9,270) was computed by similar analyses based on the polymerase proteins (gene size amounts more than 75% of the viral genome) of the NCBI reference set of human HBV genotypes A-H (File S2 and File S2c). The results support a common ancestral origin of protein X in ortho- as well as avihepadnavirus. This time point in hepadnavirus evolution marks the onset of gene inactivation of X in avihepadnavirus and the start of adaptation of X towards its present function in orthohepadnavirus. The long period of X protein evolution and the high rate of mutation in hepadnavirus genomes prevent sequence-based reconstructions of X ancestors. We therefore turned towards the tertiary structure of X and applied *in silico* modeling of protein X, because an X-ray structure is not available.

### *In silico* generation of HBx tertiary structure

**Figure 2:**
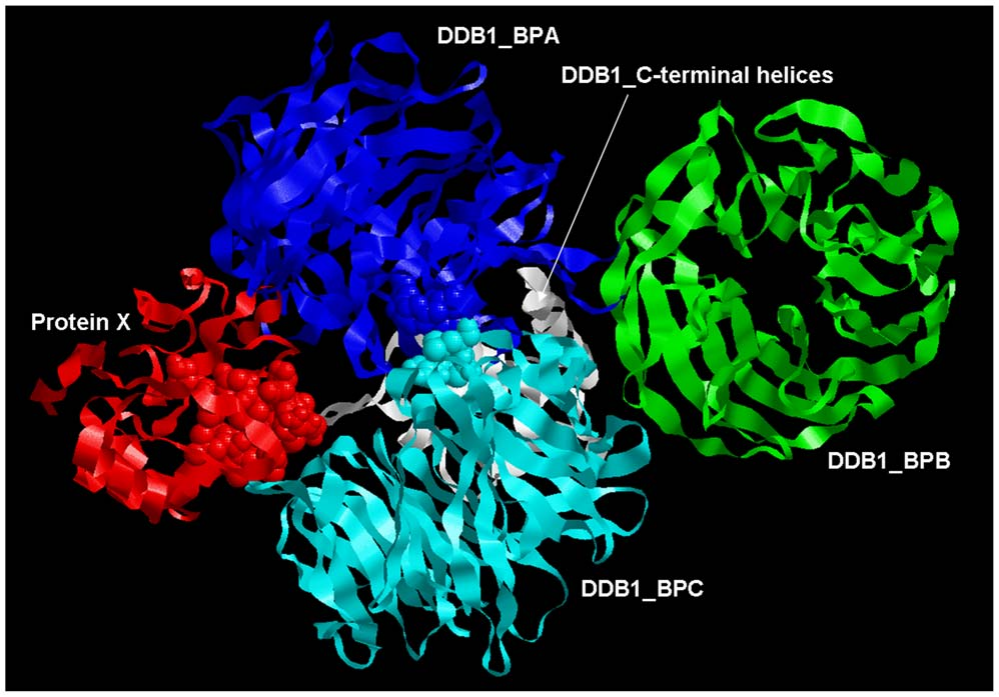
3D-model of the X-DDB1 heterodimeric complex. X protein is in red and the DDB1 domains A, B and C are in blue, green and magenta, respectively. The α-helical C-terminal part of DDB1 is white colored. The H-box residues of protein X (88–101) and the residues Arg327, Leu328, Pro358, Ala381, Phe382 and Asn1005 of DDB1_C are in spacefilled format. The mean distance between the spacefilled residues of protein X and DDB1 amounts 25 Å, approximately. The corresponding PDB coordinate file of the complex is available as File S6.

**Figure 3:**
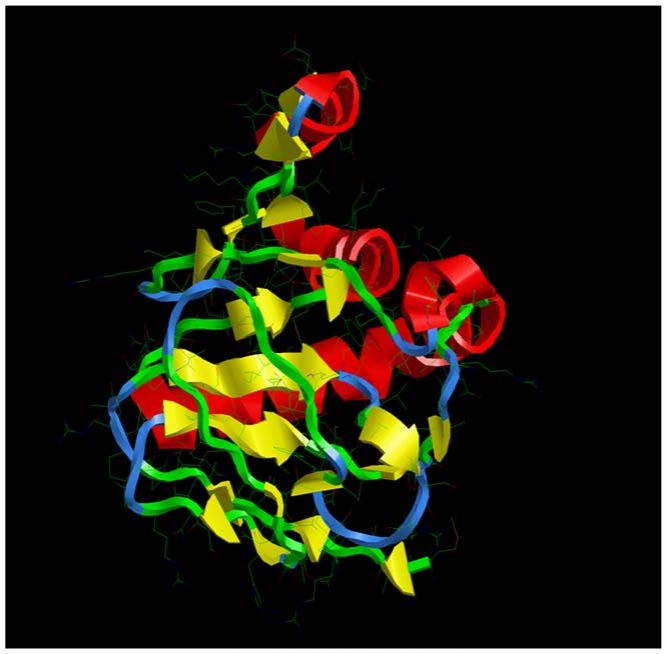
The model1 3D-structure of protein X (genotype D consensus sequence). X protein is coloured by the default RasMol script “structure” according to its secondary structure determined by the Kabsch & Sander DSSP algorithm (red, yellow, blue and green for helices, sheets, turns and others, respectively). A PDB coordinate file is provided as File S3.

An HBV type D consensus sequence of X was submitted to *i*-TASSER [27] for 3D modeling. Parent structures with similarity to protein X above the default threshold level were not found in the PDB database. Five candidate structures were obtained with C-score values in the range of −3.89 to −5.01. To verify if our X model is compatible with existing data, we performed *in silico* docking experiments with the X-binding proteins HBXIP and DDB1. As a crystal structure of HBXIP was not yet available, five models of the 3D-structure of HBXIP were generated by combined *ab initio* and homology modeling (parent structures in PDB were 1j3w, 2hz5, 1bx4A, 1v5wA, 1hdoA and 1p9vA and C-scores ranged between −3.61 and −4.58). Recently, a tertiary structure of HBXIP has been determined by means of X-ray diffraction [40] revealing a striking similarity to the *in silico* generated models 1, 2, 3 and 5, but not 4. The ClusPro docking procedure generated 10 candidate dimer models for each combination of five X with five HBXIP monomer structures [28]. Using ALASCAN, all 250 dimer models were subjected to computational alanine replacement scanning for the determination of their interface stability and composition [30]. The X/HBXIP model1/model1 heterodimer complex scores among the top 10 as judged by docking parameters and among the top 3 for interface stability. Also, the tetrapeptide ^137^CRH^140^K of X, known to be obligatory for X-HBXIP complex formation [12], was among the contact residues of the interface. The X/HBXIP model1/model1 heterodimer complex exclusively fulfilled all these conditions. PDB coordinate files of X model1 and HBXIP model1 are added as File S3 and File S4, respectively. Combined, the C-values of the monomer models, the stability of the X/HBXIP heterodimers and the composition of their interfaces indicate models 1 of both X and HBXIP as the best achievable results of 3D modeling. The 3D-structure of the DDB1 interaction partner has also been determined by X-ray diffraction (PDBid 2b5m). Similar results were obtained for the complex between the proteins X and DDB1 [41] in which case the binding nonapeptide ^91^KVLHKRTL^99^G participated in the interface, except ^92^V and ^99^G. This nonapeptide is the core sequence of the α-helical motif called H-box [13]. The 3D-structure of the H-box/DDB1 complex revealed interactions of Arg96, Leu98 and Gly99 of the H-box 13-mer peptide with residues Arg327, Leu328, Pro358, Ala381, Phe382 and Asn1005 of the BPC domain at the opening of the BPA/BPC double propeller pocket of DDB1. We performed docking of DDB1 with protein X model1. Indeed, X protein is captured by the “mouth” of the BPA/BPC double propeller pocket with the H-box α-helical motif directed towards its throat (Fig. 2). Five similar structures with slightly different orientations were present in the top 10 solutions of the docking experiments (hydrophobicity-favoured). We have selected the complex with the shortest distances between the ^α^C-atoms of the interacting residues as specified above (R96XtoR327DDB1, 23,7Å; L98XtoF382DDB1, 27.1Å; G99XtoN1005DDB1, 27.1Å). Computational replacement of H-box amino acids by alanine generally affected the complex stability for all top 10 solutions. Residues outside of the H-box motif also participate at the X/DDB1 interface. Position and orientation of the X domain in the X/DDB1 3D-model is similar to that described for X-ray structures of DDB1 complexes with the 13-mer oligopeptide of X called H-box [13] and with paramyxovirus V protein [42]. Distances between interacting residues are smaller in H-box-DDB1 complexes than in the full-length X/DDB1 complex. The rigid docking procedure applied does not account for subsequent, local rearrangements due to natural protein flexibility. A PDB coordinate file of the complex is available as File S6. On basis of these criteria, we conclude that the *in silico* generated X protein model1 (Fig. 3) is most likely to mimic the natural tertiary structure of X (genotype D consensus sequence). Nearly identical 3D-models were generated for X consensus and ancestral sequences both derived from the NCBI reference set of the human HBV genotypes A-H.

### The 3D-structure of protein X resembles that of DNA glycosylase

**Figure 4:**
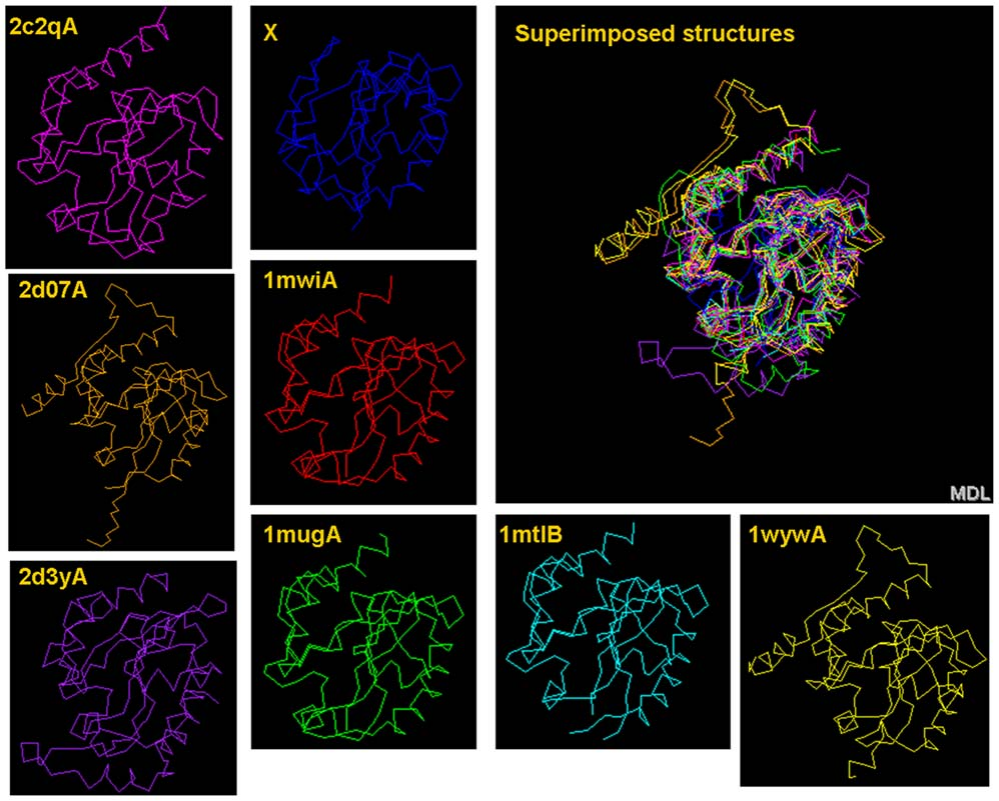
3-Dimensional similarity between protein X and DNA glycosylase. DNA glycosylases are indicated by their PDB identifier and chain indicator (A/B). A superimposition of the backbone structures is shown in the upper right panel.

Analysis of the sequence similarity of X with other sequences detects a minimum level of similarity. We therefore submitted the X 3D-structure model1 to the servers DALILITE and MATRAS querying the presence of similarly structured proteins in the PDB database. The Dali method uses a weighted sum of similarities of intra-molecular distances. MATRAS exerts protein tertiary structure comparison using a Markov transition model of evolution. Both servers identified the X tertiary structure as significantly related to the 3D-structure of DNA glycosylase (Table 1). DALILITE searched the PDB database for X similar structures and ranked all positive hits, whereas MATRAS employed its own (non-redundant) subset of PDB structures and reported positive types of structures. Consequently, the first four MATRAS hits are equivalent to the upper 17 DALILITE hits, both displaying DNA glycosylases with different nucleotide specificities. The low value for amino acid identity (%id, Table 1) illustrates the low level of sequence similarity at this significant 3D-structural similarity. In addition, the hits 18 (DALILITE) and 5 (MATRAS) mark the transition of high-to-low similarity of X protein with molecules other than DNA glycosylases. Structures of various DNA glycosylases and X protein are displayed individually in similar orientation and in overlay position (Fig. 4). In a phylogenetic tree based on sequence alignment of protein X with a collection of DNA glycosylases [14], X occupied the position of a member of the *MUG* family (File S5). However, the tree suffered from low bootstrap support and its topology was sensitive to minor rearrangements in the alignment probably due low sequence similarity.

**Table 1:** Structural relatives of HBx.

DALI Server:					
*No:*	*Chain*	*Zsc*	*Lali*	*%id*	*Description*
1	1mwiA	9.9	130	10	G/U mismatch-specific DNA glycosylase
2	1mwjA	9.8	130	10	G/U mismatch-specific DNA glycosylase
3	***1mugA***	9.7	130	10	G:T/U specific DNA glycosylase
4	1mtlB	9	125	10	G/U mismatch-specific DNA glycosylase
5	1mtlA	8.5	120	10	G/U mismatch-specific DNA glycosylase
6	***1wywA***	8.4	135	8	G/T mismatch-specific thymine DNA glycosylase
7	2c2pA	8.3	132	10	G/U mismatch-specific DNA glycosylase
8	***2c2qA***	8.2	132	8	G/U mismatch-specific DNA glycosylase
9	2d07A	7.8	133	7	G/T mismatch-specific thymine DNA glycosylase
10	1vk2A	6	119	13	Uracil DNA glycosylase TM0511
11	1l9gA	6	124	13	Uracil DNA glycosylase Thermotoga maritima
12	1ui1A	5.9	122	11	Uracil DNA glycosylase
13	1ui0A	5.7	121	9	Uracil DNA glucosylase
14	***2d3yA***	5.6	123	14	Uracil DNA glycosylase
15	2dp6A	5.5	126	13	Uracil DNA glycosylase
16	2demA	5.5	123	14	Uracil DNA glycosylase
17	2ddgA	5.4	122	15	Uracil DNA glycosylase
18	1cuwB	4.9	103	11	Cutinase

MATRAS Server:					
*No:*	*Chain*	*Zsc*	*Lali*	*%id*	*Description*
1	***1wywA***	81.09	159	6	G/T mismatch-specific thymine DNA glycosylase
2	***2c2qA***	70.73	153	9.1	G/U mismatch-specific DNA glycosylase
3	***1mugA***	59.77	121	7.8	G:T/U specific DNA glycosylase
4	***2d3yA***	51.77	159	13.6	Uracil DNA glycosylase
5	2jfnA	9.8	61	9.8	Glutamate racemase

“Chain” indicates the PDB entry (lower case) followed by the chain identifier (upper case). DALI and MATRAS results are ranked according to their Z-scores (Zsc) with threshold values of 2 and 5, respectively. “Lali” and “%id” indicate the length of the aligned polypeptide chains and the percentage of identical residues. Equal hits are in italic boldface. DALI queries apply to the entire PDB database, whereas MATRAS employs a representative portion of PDB that is updated weekly.

**Figure 5:**
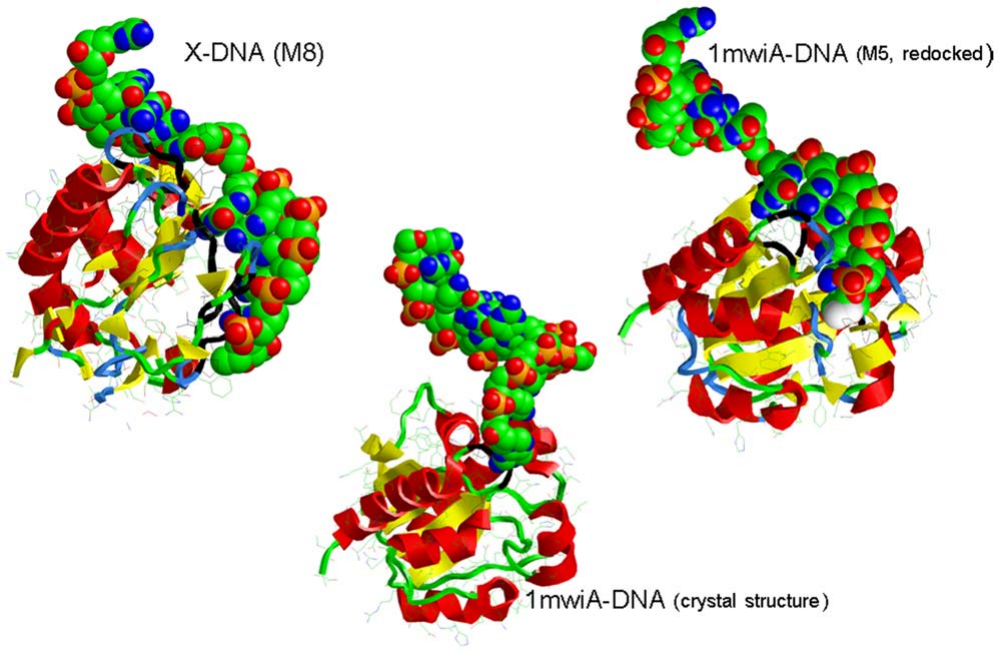
DNA-binding ability of protein X compared with E. coli MUG DNA glycosylase. The original PDB coordinate file (1mwiA-DNA, crystal structure) was divided into the two component chains A (DNA glycosylase) and D (oligoDNA). Protein-DNA docking facilitated the formation of the dimer complexes X-DNA (M8) and 1mwiA-DNA (M5, redocked). Protein and DNA moieties are displayed in 2D-cartoon and space-filling format, respectively. Black-colored aminoacid residues mark the protein-DNA interfaces (see table 2 for their specification).

DNA glycosylase proteins have been characterized by a conserved central domain with N- and C-terminal oligopeptide motifs representing critical residues of the active site. N- and C-terminal extensions present in mammalian and insect DNA glycosylase harbor SUMO-interaction and SUMOylation consensus motifs and AT-hook motifs for non-specific DNA binding capacity [14]. Bacterial family-2 DNA glycosylase (i.e. the *E. coli* representative of *MUG* DNA glycosylase, pdb entry 1mug) only consists of the conserved central domain and differs from animal (“TDG”) glycosylases by the presence of a region for non-specific DNA binding and a capacity to interact with the complementary DNA strand opposite from the damaged base [14]. The structural similarity of DNA glycosylases with protein X is confined to the conserved central domain and the flanking oligopeptide motifs including the critical catalytic asparagine required for specific glycosylase activity are absent in X. *E. coli MUG* protein in complex with a self-complementary oligodeoxynucleotide carrying an abasic moiety in its center has been studied by X-ray analysis [16], [17]. A DNA binding domain and orientation of the ligand towards the center of catalytic activity were described. We divided the PDB coordinate file of this complex (1mwi) into the two component chains (protein A and oligoDNA D). Subsequently, we constructed heterodimer complexes of protein X with chain D and redocked the chains A and D for control purposes. Indeed, considerable resemblance can be observed between X-DNA (Model8), 1mwiA-DNA (Model5, redocked) and the original 1mwiA-DNA crystal structure (Fig. 5). Also, the redocked 1mwiA-DNA model5 complex displayed the oligopeptide motifs specifying DNA glycosylase enzymatic activity in the protein/DNA interface region (Table 2). A large string of C-terminal residues in the docked complexes attends to the binding of this oligonucleotide. The absence of similarity between X and DNA glycosylase among these interface residues again underlines the importance of structure similarity compared to sequence conservation of these proteins. The relatively small protein-DNA interface in the crystal structure of 1mwiA-DNA may point to preferential selection of this orientation during the crystallization process that may be promoted by an enhanced flexibility around the few contact residues. These *in silico* results substantiate a significant relationship of X protein structure with that of the *MUG* family of DNA glycosylases. Although protein X is generally considered as devoid of DNA binding capacity, interaction of protein X with single-stranded DNA has been demonstrated by means of band-shift assays [43].

**Table 2:** Interface composition of protein-DNA complexes.

HBx-DNA		1mwiA-DNA		1mwiA-DNA	
(M8)		(M5, redocked)		(crystal structure)	
Amino acid		Amino acid		Amino acid	
Number	Name	Number	Name	Number	Name
33	PRO	**16**	**GLY**	36	ARG
34	LEU	**17**	**ILE**	82	LYS
35	GLY	***18***	***ASN***	**143**	**GLY**
36	THR	**19**	**PRO**	**144**	**LEU**
37	LEU	**20**	**GLY**	**145**	**SER**
38	SER	**21**	**LEU**	**146**	**ARG**
39	SER	22	SER		
40	PRO	23	SER		
41	SER	30	PHE		
43	SER	32	HIS		
45	VAL	34	ALA		
96	ARG	35	ASN		
115	CYS	74	THR		
116	LEU	75	VAL		
117	PHE	76	GLN		
118	LYS	77	ALA		
119	ASP	78	ASN		
120	TRP	108	GLY		
123	LEU	109	LYS		
127	ILE	110	GLN		
128	ARG	111	ALA		
130	LYS	113	GLU		
131	VAL	120	GLY		
132	PHE	121	ALA		
133	VAL	122	GLN		
134	LEU	123	TRP		
135	GLY	**139**	**PRO**		
136	GLY	**140**	**ASN**		
137	CYS	**142**	**SER**		
138	ARG	**143**	**GLY**		
139	HIS	**144**	**LEU**		
140	LYS	**145**	**SER**		
141	LEU	**146**	**ARG**		
142	VAL	147	VAL		
143	CYS				
144	ALA				
145	PRO				
147	PRO				

Amino acid numbering refers to unaligned protein sequences. Bold-faced residues indicate the oligopeptide motifs specifying DNA glycosylase enzymatic activity. The critical catalytic asparagine residue (1mwiA-DNA, ASN18) is shown in italics.

**Figure 6:**
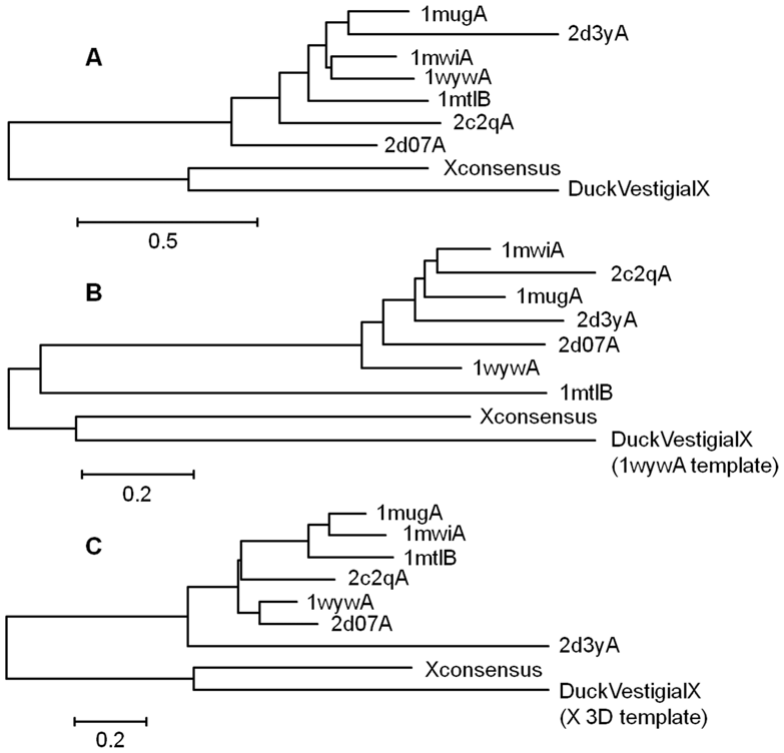
Similarity dendrograms of DNA glycosylase and X 3D-structures. Matrices of pairwise DRMS values (relative positions of ^ß^C atoms) were fed into the neighbor joining tree building facility of MEGA4. (A) After modeling of duck vestigial X (DV_HBx) without a user-defined template structure. (B) After modeling of duck vestigial X with 1wywA DNA glycosylase provided as template structure. (C) After modeling of duck vestigial X with X-consensus 3D-structure provided as template structure. Note the size difference between the scale bars A and B or C.

We investigated whether the duck hypothetical X translation product (139 amino acid residues) can be folded into a tertiary structure with similarity to human X (154 AAs in length) and hence to *MUG* DNA glycosylase. Similarity between the 3D-structures of X (human and duck hepadnaviruses) and DNA glycosylases was measured by means of the DRMS (root mean square deviation) parameter indicating the relative positions of ^β^C-atoms (first C-atom in the side chain) in the 3D-structures of the proteins under investigation. DRMS values of proteins were put into a pairwise similarity matrix. By means of neighbor-joining cluster analysis, the relative similarities could be displayed as a dendrogram. Indeed, duck vestigial X folded into a structure with similarity to human X protein model1 by means of unconstrained modeling (Fig. 6A). This similarity could further be improved by providing X model1 or DNA glycosylase (1wyw) as template structures for the modeling of duck X protein (Fig. 6B and 6C, respectively). Neither similarity nor its improvement was observed after similar comparison of the N-terminal part of duck capsid protein with human X or DNA glycosylase. Like animal X protein, avian vestigial X also displays a tertiary structure, which resembles that of DNA glycosylase. A further reconstruction of X protein phylogeny towards cellular DNA glycosylase failed due to the large difference in mutational rates between virus and host sequences (2×10^−5^ vs.1×10^−9^ r/s/y, respectively). Partitioning of the data set in BEAST reflecting this rate difference effectively reduced the time scale, but did not allow the repetitive comparison of a single amino acid replacement in DNA glycosylase with 5000 replacements in X. Our results indicate a common ancestral origin of members of the *MUG* family of DNA glycosylases and protein X of ortho- as well as avihepadnavirus.

## Discussion

The Bayesian analysis of X protein phylogeny showed that divergence of both ortho- and avihepadnavirus into the present strains started about 25,000 years ago and that their most recent common ancestor appeared to be about 125,000 years of age. Time calculations of these evolutionary events rely on the assumption of a constant rate of amino acid replacement during the entire period of evolution, of which only the recent 25 years are available for experimental verification [39]. A fixed *mean* rate of mutation does not preclude rate variation in time or locally along the sequence. Also, another fixed value for the mean rate of mutation obtained by advancing insight will proportionally alter these dates without affecting the evolutionary sequence of events. The time span of about 100,000 years between the MRCA and the onset of virus divergence may be considered as the childhood period of hepadnaviridae, during which orthohepadnavirus developed a functional protein X and avihepadnavirus evolved without X. Our results are in support of an ancestral hepadnaviral genome carrying an X sequence with orthology to the core domain of DNA glycosylase. The presence of a cellular DNA glycosylase gene in a virus genome is not unprecedented. The fully functional uracil-DNA glycosylase (*UDG*) gene of Herpes simplex virus prevents the accumulation of G:C→A:T transition mutations by means of base excision repair before replication. Family-1 (*UDG*) and family-2 (*MUG*) DNA glycosylases differ considerably by sequence, structure and repair mechanism [44]. Exchange of genomic information between virus and hosts is known to occur during the evolution of (mostly large) DNA viruses followed by selection and retention of genes that increase viral fitness [45]. The similarity between ortho- and avihepadnavirus protein X and the core domain of *MUG* DNA glycosylase suggests that an ancestral hepadnavirus might have “captured” the corresponding sequence from a host gene more than approximately 1000 centuries ago. However, avihepadnavirus replicates without X and apparently has found another solution during evolution. The host-to-virus gene transfer scenario may seem rather complicated given the extensive gene overlaps in the hepadnaviral genomes. The acquisition of an ancestral X sequence by the HBV genome may have occurred before its compression into overlapping genes. In this case, mutational rate constancy along the virus lineage of an originally cellular sequence is unlikely to occur. The availability of a *bona fide* tertiary structure of protein X promotes investigations into the pleiotropic spectrum by which X affects cellular functions like the ubiquitin ligase activity of CUL4-DDB1 E3 complexes [13], [46]. In conclusion, this study indicates an evolutionary relationship between the hepadnaviral protein X and cellular DNA glycosylase.

## Supporting Information

File S1 **BEAST xml file corresponding to** **Figure 1** **: Phylogeny and divergence time estimates of hepadnaviral protein X.**
(XML)

File S2 **Phylogeny of and divergence time estimates of human HBV genotypes A-H based on polymerase protein sequences.** (A) GenBank entries refer to the NCBI reference set of HBV, of which the polymerase amino acid sequences were used for BEAST analysis. The evolutionary sequence of events is displayed in tree format with node ages. (B) Monte Carlo Markov (MCMC) estimates and parameter statistics are given without decimal numbers for mean values and highest posterior density interval (HPD). Minor differences between corresponding numbers in A and B are due to the stochastic character of the MCMC algorithm. **File S2c. BEAST xml file corresponding to File S2: Phylogeny of and divergence time estimates of human HBV genotypes A-H based on polymerase protein sequences.**
(DOC)

File S3 **PDB coordinate file of protein X tertiary structure (HBx type D consensus sequence).**
(PDB)

File S4 **PDB coordinate file of HBXIP tertiary structure.**
(PDB)

File S5 **Evolutionary position of HBx among DNA glycosylases.** A: A phylogenetic tree of MUG proteins was constructed according to Cortazar et al. (2007, DNA Repair, 6, 489–504) with HBx (D-type consensus sequence) indicated in bold typeface. B: A similar tree (the same set of sequences with ancestor and consensus HBx derived from the NCBI reference set of HBV) was constructed after re-alignment by ProbCons (Do et al., 2005, Genome Res., 156, 2, 330–340) followed by rounds of manual refinement with special attention at gap borders.
(TIF)

File S6 **PDB coordinate file of the protein X/DDB1 3D-complex.**
(PDB)
