# Supplementary material for: Protein X of Hepatitis B Virus: Origin and Structure Similarity with the Central Domain of DNA Glycosylase
Source: PLoS One. 2011 Aug 5;6(8):e23392. doi: 10.1371/journal.pone.0023392 (PMC3153941; doi:10.1371/journal.pone.0023392)
Supplement: File S2 — Phylogeny of and divergence time estimates of human HBV genotypes A-H based on polymerase protein sequences. (A) GenBank entries refer to the NCBI reference set of HBV, of which the polymerase amino acid sequences were used for BEAST analysis. The evolutionary sequence of events is displayed in tree format with node ages. (B) Monte Carlo Markov (MCMC) estimates and parameter statistics are given without decimal numbers for mean values and highest posterior density interval (HPD). Minor differences between corresponding numbers in A and B are due to the stochastic character of the MCMC algorithm. File S2c. BEAST xml file corresponding to File S2: Phylogeny of and divergence time estimates of human HBV genotypes A-H based on polymerase protein sequences. (DOC) [file pone.0023392.s002.doc]

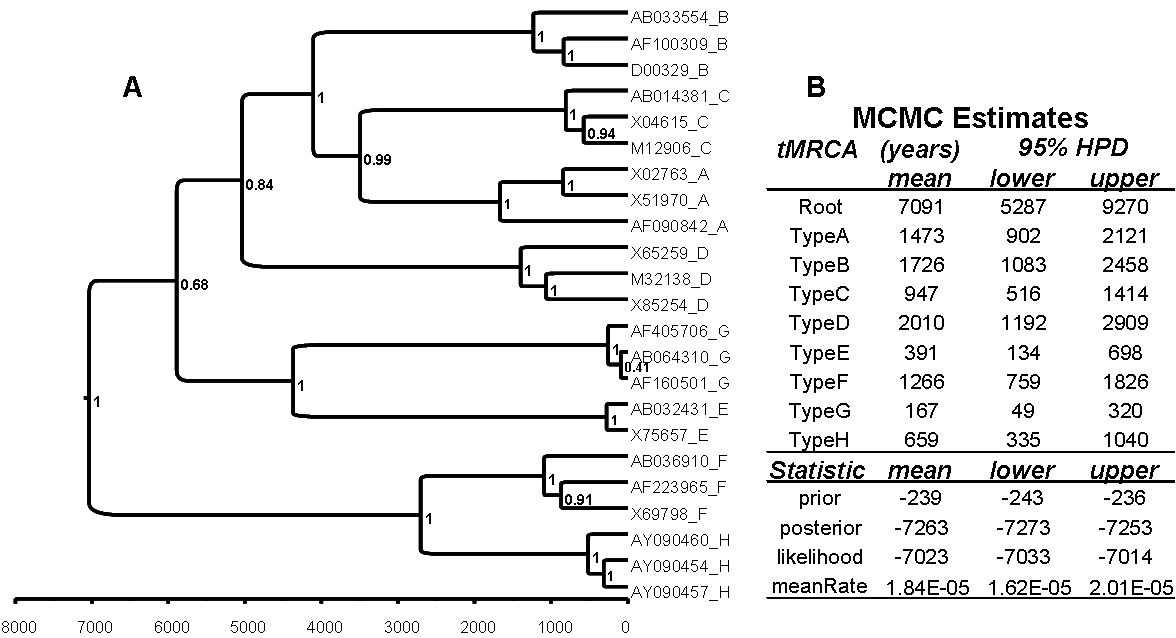


File S2 (A&B)

File S2c

<?xml version="1.0" standalone="yes"?>

<!-- Generated by BEAUTi v1.4.8 -->

<!-- by Alexei J. Drummond and Andrew Rambaut -->

<!-- Department of Computer Science, University of Auckland and -->

<!-- Institute of Evolutionary Biology, University of Edinburgh -->

<!-- http://beast.bio.ed.ac.uk/ -->

<beast>

<!-- The list of taxa analyse (can also include dates/ages). -->

<!-- ntax=23 -->

<taxa id="taxa">

<taxon id="X02763_A"/>

<taxon id="X51970_A"/>

<taxon id="AF090842_A"/>

<taxon id="AF100309_B"/>

<taxon id="AB033554_B"/>

<taxon id="M12906_C"/>

<taxon id="AB014381_C"/>

<taxon id="X65259_D"/>

<taxon id="M32138_D"/>

<taxon id="X85254_D"/>

<taxon id="AB032431_E"/>

<taxon id="X69798_F"/>

<taxon id="AB036910_F"/>

<taxon id="AF223965_F"/>

<taxon id="AF160501_G"/>

<taxon id="AB064310_G"/>

<taxon id="AF405706_G"/>

<taxon id="AY090454_H"/>

<taxon id="AY090457_H"/>

<taxon id="AY090460_H"/>

<taxon id="D00329_B"/>

<taxon id="X04615_C"/>

<taxon id="X75657_E"/>

</taxa>

<taxa id="TypeA">

<taxon idref="AF090842_A"/>

<taxon idref="X02763_A"/>

<taxon idref="X51970_A"/>

</taxa>

<taxa id="TypeB">

<taxon idref="AB033554_B"/>

<taxon idref="AF100309_B"/>

<taxon idref="D00329_B"/>

</taxa>

<taxa id="TypeC">

<taxon idref="AB014381_C"/>

<taxon idref="M12906_C"/>

<taxon idref="X04615_C"/>

</taxa>

<taxa id="TypeD">

<taxon idref="M32138_D"/>

<taxon idref="X65259_D"/>

<taxon idref="X85254_D"/>

</taxa>

<taxa id="TypeE">

<taxon idref="AB032431_E"/>

<taxon idref="X75657_E"/>

</taxa>

<taxa id="TypeF">

<taxon idref="AB036910_F"/>

<taxon idref="AF223965_F"/>

<taxon idref="X69798_F"/>

</taxa>

<taxa id="TypeG">

<taxon idref="AB064310_G"/>

<taxon idref="AF160501_G"/>

<taxon idref="AF405706_G"/>

</taxa>

<taxa id="TypeH">

<taxon idref="AY090454_H"/>

<taxon idref="AY090457_H"/>

<taxon idref="AY090460_H"/>

</taxa>

<!-- The sequence alignment (each sequence refers to a taxon above). -->

<!-- ntax=23 nchar=845 -->

<alignment id="alignment" dataType="amino acid">

<sequence>

<taxon idref="X02763_A"/>

MPLSYQHFRKLLLLDDGTEAGPLEEELPRLADADLHRRVAEDLNLGNLNVSIPWTHKVGNFTGLYSSTVPIFNPEWQTPSFPKIHLQEDIINRCQQFVGPLTVNEKRRLKLIMPARFYPTHTKYLPLDKGIKPYYPDQVVNHYFQTRHYLHTLWKAGILYKRETTRSASFCGSPYSWEQELQHGRLVIKTSQRHGDESFCSQSSGILSRSSVGPCIRSQLKQSRLGLQPRQGRLASSQPSRSGSIRAKAHPSTRRYFGVEPSGSGHIDHSVNNSSSCLHQSAVRKAAYSHLSTSKRQSSSGHAVEFHCLPPNSAGSQSQGSVSSCWWLQFRNSKPCSEYCLSHLVNLREDWGPCDEHGEHHIRIPRTPARVTGGVFLVDKNPHNTAESRLVVDFSQFSRGISRVSWPKFAVPNLQSLTNLLSSNLSWLSLDVSAAFYHIPLHPAAMPHLLIGSSGLSRYVARLSSNSRINNNQYGTMQNLHDSCSRQLYVSLMLLYKTYGWKLHLYSHPIVLGFRKIPMGVGLSPFLLAQFTSAICSVVRRAFPHCLAFSYMDDVVLGAKSVQHRESLYTAVTNFLLSLGIHLNPNKTKRWGYSLNFMGYIIGSWGTLPQDHIVQKIKHCFRKLPVNRPIDWKVCQRIVGLLGFAAPFTQCGYPALMPLYACIQAKQAFTFSPTYKAFLSKQYMNLYPVARQRPGLCQVFADATPTGWGLAIGHQRMRGTFVAPLPIHTAELLAACFARSRSGAKLIGTDNSVVLSRKYTSFPWLLGCTANWILRGTSFVYVPSALNPADDPSRGRLGLSRPLLRLPFQPTTGRTSLYAVSPSVPSHLPVRVHFASPLHVAWRPP

</sequence>

<sequence>

<taxon idref="X51970_A"/>

MPLSYQHFRKLLLLDDGTEAGPLEEELPRLADADLNRRVAEDLNLGNLNVSIPWTHKVGNFTGLYSSTAPIFNPEWQTPSFPKIHLQEDIINRCQQFVGPLTVNEKRRLKLIMPARFYPTHTKYLPLDKGIKPYYPDQVVNHYFQTRHYLHTLWKAGILYKRETTRSASFCGSPYSWEQELQHGRLVIKTSQRHGDESFCSQPSGILSRSSVGPCIRSQLKQSRLGLQPHQGPLASSQPGRSGSIRARVHPSTRRCFGVEPSGSGHVDPSVNNSSSCLRQSAVRKAAYSHLSTSKRQSSSGHAVEFHCLPPSSARPQSQGSVFSCWWLQFRNSKPCSEYCLSHLVNLREDRGPCDEHGEHHIRIPRTPARVTGGVFLVDKNPHNTAESRLVVDFSQFSRGITRVSWPKFAIPNLQSLTNLLSSNLSWLSLDVSAAFYHIPLHPAAMPHLLIGSSGLSRYVARLSSNSRINNNQYGTMQNLHDSCSRQLYVSLMLLYKTYGWKLHLYSHPIVLGFRKIPMGVGLSPFLLAQFTSAICSVVRRAFPHCLAFSYMDDVVLGAKSVQHREFLYTAVTNFLLSLGIHLNPNKTKRWGYSLNFMGYVIGSWGTLPQDHIVQKIKHCFRKLPVNRPIDWKVCQRIVGLLGFAAPFTQCGYPALMPLYACIQAKQAFTFSPTYKAFLSKQYMNLYPVARQRPGLCQVFADATPTGWGLAIGHQRMRGTFVAPLPIHTAELLAACFARSRSGAKLIGTDNSVVLSRKYTSFPWLLGCAANWILRGTSFVYVPSALNPADDPSRGRLGLSRPLLRLPFQPTTGRTSLYAVSPSVPSHLPVRVHFASPLHVAWRPP

</sequence>

<sequence>

<taxon idref="AF090842_A"/>

MPLSYQHFRKLLLLDDETEAGPLEEELPRLADEDLNRRVAEDLNLGNLNVSIPWTHKVGNFTGLYSSTVPIFNPEWQTPSFPKIHLHEDIANRCQQFVGPLTVNEKRRLKLIMPARFYPNSTKYLPLDKGIKPYYPDHVVNHYFQTRHYLHTLWKAGILYKRETTRSASFCGSPYSWEQELHHGRLVIKTSQRHGDEPFCSQPSGILSRSSVGPCIRSQFKQSRLGLQPHQGPLATSQPGRSGSIRARVHSPTRRCFGVEPSGSGHIGHSASSASSCLHQSAVRKAAYSHLSTSKRQSSSGHAVEFHSFPPSSARSQSQGPVFSCWWLQFRNTQPCSNYCLSHLVNLLEDWGPCAEHGEHHIRIPRTPARVTGGVFLVDKNPHNTAESRLVVDFSQFSRGITRVSWPKFAVPNLQSLTNLLSSNLSWLSLDVSAAFYHIPLHPAAMPHLLIGSSGLSRYVARLSSNSRINNNQHGTLQNLHDSCSRQLYVSLMLLYKTYGWKLHLYSHPIILGFRKIPMGVGLSPFLLAQFTSAICSVVRRAFPHCLAFSYMDDVVLGAKSVQHLESLYTGVTNFLLSLGIHLNPNKTKRWGYSLNFMGYVIGSWGTLPQDHIVQKIKHCFRKLPVNRPIDWKVCQRIVGLLGFAAPFTQCGYPALMPLYACIQAKQAFTFSPTYKAFLSKQYMNLYPVARQRPGLCQVFADATPTGWGLAIGHQRMRGTFVAPLPIHTAELLAACFARSRSGAKLIGTDNSVVLSRKYTSFPWLLGCTANWILRGTSFVYVPSALNPADDPSRGRLGLYRPLLRLPYRPTTGRTSLYAVSPSVPSHLPVRVHFASPLHVAWRPP

</sequence>

<sequence>

<taxon idref="AF100309_B"/>

MPLSYQHFRKLLLLDE--EAGPLEEELPRLADEGLNRRVAEDLNLGNLNVSIPWTHKVGNFTGLYSSTVPCFNPKGQTPSFPDIHLQEDIVDRCKQFVGPLTVNENRRLKLIMPARFYPNVTKYLPLDKGIKPYYPEYVVDHYFQTRHYLHTLWKAGILYKRESTRSASFCGSPYSWEQDLQHGRLVFQTSKRHGDKSFCPQSPGILPRSSVGPCIQSQLRKSRLGPQPAQGQLAGRQQGGSGSIRAGVHPSPWGTVGVEPSGSGPTHNCANSASSCLHQSAVRKAAYSLISTSKGHSSSGRAVELHHFPPNSSRSQSQGPVLSCWWLQFRNSEPCSEYCLCHIVNLIEDWGPCTEHGEHRIRTPRTPARVTGGVFLVDKNPHNTTESRLVVDFSQFSRGNTRVSWPKFAVPNLQSLTNLLSSNLSWLSLDVSAAFYHLPLHPAAMPHLLVGSSGLSRYVARLSSNSRIINNQHRTMQNLHNSCSRNLYVSLMLLYKTYGRKLHLYSHPIILGFRKIPMGVGLSPFLLAQFTSAICSVVRRAFPHCLAFSYMDDVVLGAKSVQHLESLYAAVTNFLLSLGIHLNPHKTKRWGYSLNFMGYVIGSWGTLPQEHIVQKIKLCFRKLPVNRPIDWKVCQRIVGLLGFAAPFTQCGYPALMPLYACIQAKQAFTFSPTYKAFLSKQYLNLYPVARQRPGLCQVFADATPTGWGLAIGHQRMRGTFLSPLPIHTAELLAACFARSRSGAKLIGTDNSVVLSRKYTSFPWLLGCAANWILRGTSFVYVPSALNPADDPSRGRLGLYRPLLRLFYRPTTGRTSLYADSPSVPSHLPDRVHFASPLHVAWRPP

</sequence>

<sequence>

<taxon idref="AB033554_B"/>

MPLSYQHFRKLLLLDD--EAGPLEEELPRLADEGLNRRVAEDLNLGNLNVSIPWTHKVGNFTGLYSSTVPSFNPQWQTPSFPDIHLQEDIIDKCKQFVGPLTVNENRRLKLIMPARFYPNVTKYLPLDKGIKPYYPEHVVNHYFQTRHYLHTLWKAGILYKRETTRSASFCGSPYSWEQELQHGRLVLQTSKRHGDKSFRPQSSGILSRSPVGPCIQSQLRQSRLGPQPTQGQLAGLQQGGSGSIRAGIHSTPWGTVGVEPSSSGHTHNCANSSSSCLHQSAVRKEAYSPVSTSKRHSSSGNAVELHHVPPNSSRSQSQGSVLSCWWLQFRNSKPCSEHCLFHIVNLIDDWGPCAEHGEHRIRTPRTPARVTGGVFLVDKNPHNTTESRLVVDFSQFSRGNTRVSWPKFAVPNLQSLTNLLSSDLSWLSLDVSAAFYHLPLHPAAMPHLLVGSSGLSRYVARLSSNSRIINHQHRTMQNLHDSCSRNLYVSLMLLYKTYGRKLHLYSHPIILGFRKIPMGVGLSPFLLAQFTSAICSVVRRAFPHCLAFSYMDDVVLGAKSVQHLESLYAAVTNFLLSLGIHLNPQKTKRWGYSLNFMGYVIGSWGTLPQEHIVLKIKQCFRKLPVNRPIDWKVCQRIVGLLGFAAPFTQCGYPALMPLYACIQAKQAFTFSPTYKAFLHKQYLNLYPVARQRPGLCQVFADATPTGWGLAIGHQRMRGTFVSPLPIHTAELLAACFARSRSGAKLIGTDNSVVLSRKYTSFPWLLGCAANWILRGTSFVYVPSALNPADDPSRGRLGLYRPLLRLPYRPTTGRTSLYADSPSVPSHLPDRVHFASPLHVAWRPP

</sequence>

<sequence>

<taxon idref="M12906_C"/>

MPLSYQHFRKLLLLDD--EAGPLEEELPRLADEGLNRRVAEDLNLGNLNVSIPWTHKVGNFTGLYSSTVPVLNPESQTPSFPNIHLQEDIINRCQQYVGPLTVNEKRRLKLIMPARFYPNLTKYLPLDKGIKPYYPEHAVNHYFKTRHYLHTLWKAGILYKRETTRSASFCGSPYSWEQELRHGRLVFQTSTRHGDESFCSQSSGILSRSPVGPCVRSQLKQSRLGLQPQQGSLARGNQGRSGRLRARVHPTTRRSFGVEPSGSGHIDNSASSASSCFHQSAVRKTAYSHLSTSKRQSSSGHAVELHNIPPSSARSQSEGPIFSCWWLQFRNSKPCSDYCLTHIVNLLEDWGPCTEHGEHNIRIPRTPARVTGGVFLVDKNPHNTTESRLVVDFSQFSRGSTHVSWPKFAVPNLQSLTNLLSSNLSWLSLDVSAAFYHIPLHPAAMPHLLVGSSGLPRYVARLSSTSRNINYQHGTMQDLHDSCSRNLYVSLLLLYKTFGRKLHLYSHPIILGFRKIPMGVGLSPFLLAQFTSAICSVVRRAFPHCLAFSYMDDVVLGAKSVQHLESLFTSITNFLLSLGIHLNPNKTKRWGYSLNFMGYVIGSWGTLPQEHIVLKLKQCFRKLPVNRPIDWKVCQRIVGLLGFAAPFTQCGYPALMPLYACIQSKQAFTFSPTYKAFLCQQYLHLYPVARQRSGLCQVFADATPTGWGLAIGHRRMRGTFVVPLPIHTAELLAACFARDRSGAKLIGTDNSVVLSRKYTSFPWLLGCAANWILRGTSFVYVPSALNPADDPSRGRLGLYRPLLSLPFQPTTGRTSLYAVSPSVPSHLPDRVHFASPLHVAWRPP

</sequence>

<sequence>

<taxon idref="AB014381_C"/>

MPLSYQHFRKLLLLDD--EAGPLEEELPRLADEGLNRRVAEDLNLGNLNVSIPWTHKVGNFTGLYSSTVPVFNPEWQTPSFPHIHLQEDIINRCQQYVGPLTVNEKRRLKLIMPARFYPNLTKYLPLDKGIKPYYPEHAVNHYFKTRHYLHTLWKAGILYKRETTRSASFCGSPYSWEQELQHGRLVFQTSTRHGDKSFCSQSSGILSRSPVGPCVRSQLKQSRLGLQPQQGSLARGKSGRSGSIRARVHPTTRRSFGVEPSGSGHIDNSASSTSSCLHQSAVRKTAYSHLSTSKRQSSSGHAVELHHIPPSSATPQSKGPILSCWWLQFRNSKPCSDYCLSHIVNLLEDWGPCTEHGEHNIRIPRTPARVTGGVFLVDKNPHNTTESRLVVDFSQFSRGSTHVSWPKFAVPNLQSLTNLLSSNLSWLSLDVSAAFYHIPLHPAAMPHLLVGSSGLPRYVARLSSTSRNINYQHGTMQDLHDSCSRHLYVSLLLLYKTFGRKLHLYSHPIILGFRKIPMGVGLSPFLLAQFTSAICSVVRRAFPHCLAFSYMDDVVLGAKSVQHLESLFTAVTNFLLSLGIHLNPNKTKRWGYSLNFMGYVIGSWGTLPQDHIVLKIKQCFRKLPVNRPIDWKVCQRIVGLLGFAAPFTQCGYPALMPLYACIQSKQAFTFSPTYKAFLCKQYLNLYPVARQRSGLCQVFADATPTGWGLAIGHRRMRGTFVAPLPIHTAELLAACFARSRSGAKLIGTDNSVVLSRKYTSFPWLLGCAANWILRGTSFVYVPSALNPADDPSRGRLGLYRPLLHLPFRPTTGRTSLYAVSPSVPSHLPDRVHFASPLHVAWRPP

</sequence>

<sequence>

<taxon idref="X65259_D"/>

MPLSYQHFRRLLLLHD--EAGPLEEELPRLPDQGLNRRVAEDLNLGNLNVSIPWTHKVGNFTGLYSSTVPVFNPHWKTPSFPNIHLHQDIIKKCEQFVGPLTVNEKRRLQLIMPARFYPNVTKYLPLDKGIKPYYPEHLVNHYFQTRHYLHTLWKAGILYKRETTHSASFCGSPYSWEQELQHG-----------AESFHQQSSGILSRPPVGSSLQSKHRKSRLGLQSQQGHLARRQQGRSWSIRAGFHPTARRSFGVEPSGSGHTTYRASKSASCLYQSPVRKAAYPSVSTFEKHSSSGHAVELHNLPPNSARSQSERPVFPCWWLQFRNSKPCSDYCLSLIVNLREDWGPCTEHGEHHIRIPRTPARVTGGVFLVDKNPHNTAESRLVVDFSQFSRGNYRVSWPKFAVPNLQSLTNLLSSNLSWLSLDVSAAFYHLPLHPAAMPHLLVGSSGLSRYVARLSSNSRIFNNQHGTMQNLHDSCSRNLYVSLLLLYQTFGRKLHLYSHPIILGFRKIPMGVGLSPFLLAQFTSAICSVVRRAFPHCLAFSYMDDVVLGAKSVQHLESLFTAVTNFLLSLGIHLNPNKTKRWGYSLNFMGYIIGSWGTLPQDHIVQKIKECFRKLPVNRPIDWKVWQRIVGLLGFAAPFTQCGYPALMPLYACIQAKQAFTFSPTYKAFLSKQYMNLYPVARQRPGLCQVFADATPTGWGLAIGNQRMRGTIVAPLPIHTAELLAACFARSRSGAKLIGTDNSVVLSRKYTSFPWLLGCTANWILRGTSFVYVPSALNPADDPSRGRLGLSRPLLRLPFQPTTGRTSLYAVSPSVPSHLPVRVHFASPLHIAWRPP

</sequence>

<sequence>

<taxon idref="M32138_D"/>

MPLSYQHFRRLLLLDD--EAGPLEEELPRLADEGLNRHVAEELNLGNLNVSIPWTHKVGNFTGLYSSTVPVFNPHWKTPSFPNIHLHQDIIKKCEQFVGPLTVNEKRRLQLIMPARFYPKVTKYLPLDKGIKPYYPEHLVNHYFQTRHYLHTLWKAGVLYKRETTHSASFCGSPYSWEQELQHG-----------AESFHQQSSGILSRPPVGSSLQSKHCKSRLGLQSQQGLLARRQQGRSWSIRAGIHPTARRPFGVEPSGSGHTTNLASKSASCLHQSPVRKATYPSVSTFEKHSSSGHAVELHNLPPNSARSQSERPVSPCWWLQFRNSKPCSDYCLSHIVNLLEDWGPCAEHGEHHIRIPRTPARVTGGVFLVDKNPHNTEESRLVVDFSQFSRGNHRVSWPKFAVPNLQSLTNLLSSNLSWLSLDVSAAFYHLPLHPAAMPHLLVGSSGLSRYVARLSSDSRIFNHQHGTMQNLHDSCSRNLYVSLLLLYQTFGRKLHLYSHPIILGFRKIPMGVGLSPFLLAQFTSAICSVVRRAFPHCLAFSYMDDVVLGAKTVHHLESLFTAVTNFLLSLGIHLNPNKTKRWGYSLHFMGYVIGCYGSLPQDHIIQKIKECFRKLPVNRPIDWKVCQRIVGLLGFAAPFTQCGYPALMPLYACIQSKQAFTFSPTYKAFLCKQYLNLYPVARQRPGLCQVFADATPTGWGLVMGHQRMRGTFQAPLPIHTAELLAACFARSRSGANILGTDNSVVLSRKYTSFPWLLGCAANWILRGTSFVYVPSALNPADDPSRGRLGLSRPLLRLPFRPTTGRTSLYADSPSVPSHLPDRVHFASPLHVAWRPP

</sequence>

<sequence>

<taxon idref="X85254_D"/>

MPLSYQHFRRLLLLDD--DAGPLEEELPRLADEGLNRRVAEDLNLGNLNVSIPWTHKVGNFTGLYSSTVPVFNPHWKTPSFPNIHLHQDIIKKCEQFVGPLTVNEKRRLQLIMPARFYPNVTKYLPLDKGIKPYYPEHLVNHYFQTRHYLHTLWKAGILYKRETTHSASFCGSPYSWEQDLQHG-----------AESFHQQSSGILSRPPVGSSLQSKHRKSRLGLQSQQGHLARCQQGRSWSIRAGFHPTARRPFGMEPSGSGHTTNFASKSASCLHQSPVRQAAYPADSTFEKHSSSGHAVELHNLPTHSARSQSERPVSPCWWLQFRNSKPCSDHCLSLIVNLLEDWGPCAEHGEHHIRIPRTPSRVTGGVFLVDKNPHNTAESRLVVDFSQFSRGNYRVSWPKFAVPNLQSLTNLLSSNLSWLSLDVSAAFYHLPLHPAAMPHLLVGSSGLSRYVARLSSNSRILNHQHGTMPHLHDSCSRNLYVSLLLLYQTFGRKLHLYSHPIILGFRKIPMGVGLSPFLLAQFTSAICSVVRRAFPHCLAFSYMDDVVLGAKSVQHLESLFTAVTNFLLSLGIHLNPNKTKRWGYSLNFMGYIIGCHGSLPQEHIVQKIKECFRKLPINRPIDWKVCQRIVGLLGFAAPFTQCGYPALMPLYACIQSKQAFTFSSTYKAFLCKQYLNLYPVARQRPGLCQVFADATPTGWGLAMGHQRMRGTFLAPLPIHTAELLAACFARSRSGANIIGTDNSVVLSRKYTSFPWLLGCAANWILRGTSFVYVPSALNPADDPSRGRLGLSRPLLRLPFRPTTGRTSLYADSPSVPSHLPDRVHLGSPLHVAWRPP

</sequence>

<sequence>

<taxon idref="AB032431_E"/>

MPLSYQHFRRILLLDE--EAGPLEEELPRLADEDLNRRVAEDLNLQLPNVSIPWTHKVGNFTGLYSSTIPVFNPNWKTPSFPDIHLHQDIINKCEQFVGPLTVNEKRRLNLVMPARFFPISTKYLPLEKGIKPYYPDNVVNHYFQTRHYLHTLWKAGILYKRETTRSASFCGSPYSWEQELHHG-AFLDGPSRMGEESFHHQSSGIFSRPPVGSSIQSKHQKSRLGPQSQQRPLDGSQQGRSGSIRAGVHSPTRRPFGVEPSGSRHAKNIASRSASCLHQSAVRKAAYPNHSTFERHSSSGHAVEFHNISPSSAGSQSKRPVFSCWWLQFRNSEPCSDYCLTHLVNLLEDWGPCTEHGKHHIRIPRTPARITGGVFLVDKNPHNTAESRLVVDFSQFSRGSSRVSWPKFAVPNLQSLTNLLSSNLSWLSLDVSAAFYHLPLHPAAMPHLLVGSSGLSRYVARLSSNSRIINHQYGTLPNLHDSCSRNLYVSLMLLFKTFGRKLHLYSHPIIMGFRKIPMGVGLSPFLLAQFTSAICSVVRRAFPHCLAFSYMDDVVLGAKSVQHLESLYTSVTNFLLSLGIHLNPNKTKRWGYSLNFMGYVIGSWGSLPQEHIIMKIKDCFRKLPVNRPIDWKVCQRIVGLLGFAAPFTQCGYPALMPLYACIQSKQAFTFSPTYKAFLCKQYLNLYPVARQRPGLCQVFADATPTGWGLAIGHQRMRGTFMAPLPIHTAELLAACFARSRSGAKLIGTDNSVVLSRKYTSFPWLLGCAANWILRGTSFVYVPSALNPADDPSRGRLGIYRPLLRLPFQPSTGRTSLYAVSPSVPSHLPDRVHFASPLHVAWRPP

</sequence>

<sequence>

<taxon idref="X69798_F"/>

MPLSYPHFRKLLLLDD--EAGPLEEELPRLADEDLNRRVAADLNLQLPNVSIPWTHKVGNFTGLYSSTVPAFNPNWSTPSFPDIHLHQDLISKCEQFVGPLTKNELRRLKLVMPARFYPKVTKYFPMDKGIKPYYPEHAVNHYFKTRHYLHTLWKAGILYKRESTRSASFCGSPYSWEQELQHGSTSLNDTKRHGTESLCAQSSGILSRPSAGSAIQSKFQQSRLGLQHKQGQLANGKQGRSGRLRSRVHTPTRWPAGVEPSSTRCVNNLASRSASCFHQSAVREKANPSLSTSKRHTSTGNAVELNPVPPSSVGSQGKGSVLPCWWLQFRDTEPCSDYCLSHIINLLEDWGPCYEHGQHYIRTPRTPARVTGGVFLVDKNPHNTTESRLVVDFSQFSRGTTRVSWPKFAVPNLQSLTNLLSSNLSWLSLDVSAAFYHLPLHPAAMPHLLVGSSGLSRYVARLSSTSRIHDHQHGTLQNLHNSCTRNLYVSLLLLFQTLGRKLHLYSHPIILGFRKIPMGVGLSPFLLAQFTSAICSVVRRAFPHCLAFSYMDDLVLGAKSVQHLESLYTAVTNFLLSVGIHLNTSKTKRWGYSLHFMGYVIGSWGSLPQDHIVHKIKECFRKLPVNRPIDWKVCQRIVGLLGFAAPFTQCGYPALMPLYACITAKQAFVFSPTYKAFLCKQYMNLYPVARQRPGLCQVFADATPTGWGLAIGHQRMRGTFVAPLPIHTAELLAACFARSRSGATLIGTDNSVVLSRKYTSFPWLLGCAANWILRGTSFVYVPSALNPADDPSRGRLGLYRPLLRLPFQPTTGRTSLYADSPSVPSHLPDRVHFASPLHVAWRPP

</sequence>

<sequence>

<taxon idref="AB036910_F"/>

MPLSYPHFRKLLLLDD--EAGPLEEELPRLADEGLNRRVAEDLNLQLPNVSIPWTHKVGNFTGLYSSTVPAFNPNWLTPSFPDIHLHQDLISKCEQFVGPLTKNELRRLKLVMPARFYPKVTKYFPMEKGIKPYYPEHAVNHYFKTRHYLHTLWKAGILYKRESTRSASFCGSPYSWEQELQHGSTSLNDKKGHGTESFCAQSSGLLARPSAGSAIQSKFQQSRLGLQHKQGQLANGKQGRSGRLRSRVHTPSRWPAGVEPSGTGCFNNLASRSASCFHQSAVREKANPSLSTSKRHTSTGHAVELNSVPPGSVGSEGKGSVFSCWWLQFRDTEPCSDYCLSHIINLLEDWGPCHEHGEHHIRTPRTPARVTGGVFLVDKNPHNTTESRLVVDFSQFSRGTTRVSWPKFAVPNLQSLTNLLSSNLSWLSLDVSAAFYHLPLHPAAMPHLLVGSSGLPRYVARLSSTSRIHDHQHGTMQNLHNSCSRNLYVSLLLLFQTLGRKLHLYSHPIILGFRKIPMGVGLSPFLLAQFTSAICSVVRRAFPHCLAFSYMDDLVLGAKSVQHLESLYTAVTNFLLSVGIHLNTAKTKRWGYTLHFMGYVIGSWGTLPQDHIIHKIKDCFRKLPVNRPIDWKVCQRIVGLLGFAAPFTQCGYPALMPLYTCITAKQAFVFSPTYKAFLCKQYMNLYPVARQRPGLCQVFADATPTGWGLAIGHQRMRGTFVAPLPIHTAELLAACFARSRSGANIIGTDNSVVLSRKYTSFPWLLGCAANWILRGTSFVYVPSALNPADDPSRGRLGLYRPLLRLPFQPTTGRTSLYADSPSVPSHLPDRVHFASPLHVAWRPP

</sequence>

<sequence>

<taxon idref="AF223965_F"/>

MPLSYPHFRKLLLLDD--EAGPLEEELPRLADEGLNRRVAEDLNLQLPNVSIPWTHKVGNFTGLYSSTVPAFNPHWLTPSFPDIHLHQDLISKCEQFVGPLTKNELRRLKLIMPARFFPKLTKYFPLEKGIKPYYPEHAVNHYFKTRHYLHTLWKAGILYKRESTRSASFCGSPYSWEQELQHGSTSLNDKKGHGTESLCAQSTGILSRTSAGSSFQSKFQQSRLGLQQKQGHLANGKQGRSGRLRSRVHTPTRWPVGVEPSGTRCSNNLASRSASCFHQSAVREEANPSLSTSKRHTSTGNAVELNPVPPGLVGSEGKGSVFSCWWLQFRDAEPCSDYCLSHIINLLEDWGPCYEHGQHHIRTPRTPARVTGGVFLVDKNPHNTTESRLVVDFSQFSRGTTRVSWPKFAVPNLQSLTNLLSSNLSWLSLDVSAAFYHLPLHPAAMPHLLVGSSGLSRYVARLSSNSRIYDHQHGTMQNLHNSCSRNLYVSLLLLFQTLGRKLHLYSHPIILGFRKIPMGVGLSPFLLAQFTSAICSVVRRAFPHCLAFSYMDDLVLGAKSVQHLESLYTAVTNFLLSVGIHLNTSKTKRWGYNLHFMGYVIGSWGALPQDHIVHKIKECFRKLPVNRPIDWKVCQRIVGLLGFAAPFTQCGYPALMPLYTCITAKQAFVFSPTYKAFLCKQYMNLYPVARQRPGLCQVFADATPTGWGLAIGHQRMRGTFVAPLPIHTAELLAACFARSRSGANIIGTDNSVVLSRKYTSFPWLLGCAANWILRGTSFVYVPSALNPADDPSRGRLGLYRPLLRLPFQPTTGRTSLYADSPSVPSHLPVRVHFASPLHVAWRPP

</sequence>

<sequence>

<taxon idref="AF160501_G"/>

MPLSYQHFRRLLLLDE--EAGPLEEELPRLADEDLNRRVAEDLHLQLPNVSIPWTHKVGNFTGLYSSTIPVFNPDWQTPSFPNIHLHQDIITKCEQFVGPLTVNEKRRLKLVMPARFFPNSTKYLPLDKGIKPYYPENVVNHYFQTRHYLHTLWKAGILYKRETSRSASFCGSPYTWEQDLQHG-AFLDGPSRVGKEPFRQQSSRIPSRSPVGPSIQSKYQQSRLGLQSQKGPLARGQQGRSWSLWTRVHPSTRRPFGVEPSVSGHTNNFASRSASCLHQSSVREAAYSHLSTTKRQSSSGHAVELYSIPPSSTKSQSQGPVFSCWWLQFRDSEPCSDYCLSHLVNLLQDWGPCTEHGEHHIRIPRTPARVTGGVFLVDKNPHNTAESRLVVDFSQFSRGSARVSWPKFAVPNLQSLTNLLSSNLSWLSLDVSAAFYHIPLHPAAMPHLLVGSSGLSRYVARLSSDSRILDHQYGTLQNLHDSCSRQLYVSLMLLYKTFGRKLHLYSHPIILGFRKIPMGVGLSPFLLAQFTSAICSVVRRAFPHCLAFSYMDDVVLGAKSVQHLESLYTAVTNFLLSLGIHLNPNKTKRWGYSLNFMGYVIGSWGTLPQEHITQKIKQCFRKLPVNRPIDWKVCQRITGLLGFAAPFTQCGYPALMPLYACIQAKQAFTFSPTYKAFLCKQYMNLYPVARQRPGLCQVFADATPTGWGLAIGHQRMRGTFVAPLPIHTAELLAACFARSRSGAKLIGTDNSVVLSRKYTSFPWLLGCAANWILRGTSFVYVPSALNPADDPSRGRLGLCRPLLRLPFLPTTGRTSLYAVSPSVPSHLPDRVHFASPLHVTWKPP

</sequence>

<sequence>

<taxon idref="AB064310_G"/>

MPLSYQHFRRLLLLDE--EAGPLEEELPRLADEDLNRRVAEDLHLQLPNVSIPWTHKVGNFTGLYSSTIPVFNPDWQTPSFPNIHLHQDIITKCEQFVGPLTVNEKRRLKLVMPARFFPNSTKYLPLDKGIKPYYPENVVNHYFQTRHYLHTLWKAGILYKRETSRSASFCGSPYTWEQDLQHG-AFLDGPSRVGKEPFHQQSSRIPSRSPVGPSIQSKYQQSRLGLQSQKGPLARGQQGRSWSLWTRVHPSTRRPFGVEPSVSGHTNNFASRSASCLHQSSVREAAYSHLSTTKRQSSSGPAVELYSIPPSSTKSQSQGPVFSCWWLQFRDSEPCSDYCLSHLVNLLQDWGPCTEYGEHHIRIPRTPARVTGGVFLVDKNPHNTAESRLVVDFSQFSRGSARVSWPKFAVPNLQSLTNLLSSNLSWLSLDVSAAFYHIPLHPAAMPHLLVGSSGLSRYVARLSSDSRILDHQYGTLQNLHDSCSRQLYVSLMLLYKTFGRKLHLYSHPIILGFRKIPMGVGLSPFLLAQFTSAICSVVRRAFPHCLAFSYMDDVVLGAKSVQHLESLYTAVTNFLLSLGIHLNPNKTKRWGYSLNFMGYVIGSWGTLPQEHITQKIKQCFRKLPVNRPIDWKVCQRITGLLGFAAPFTQCGYPALMPLYACIQAKQAFTFSPTYKAFLCKQYMNLYPVARQRPGLCQVFADATPTGWGLAIGHQRMRGTFVAPLPIHTAELLAACFARSRSGAKLIGTDNSVVLSRKYTSFPWLLGCAANWILRGTSFVYVPSALNPADDPSRGRLGLCRPLLRLPFLPTTGRTSLYAVSPSVPSHLPDRVHFASPLHVTWKPP

</sequence>

<sequence>

<taxon idref="AF405706_G"/>

MPLSYQHFRRLLLLDE--EAGPLEEELPRLADEDLNRRVAEDLHLQLPNVSIPWTHKVGNFTGLYSSTIPVFNPDWQTPSFPNIHLHQDIITKCEQFVGPLTVNEKRRLKLVMPARFFPNSTKYLPLDKGIKPYYPENVVNHYFQTRHYLHTLWKAGILYKRETSRSASFCGSPYTWEQDLQHG-AFLDGPSRVGKEPFHQQSSRIPSRSPVGPSIQSKYQQSRLGLQSQKGPLARGQQGRSWSLWTRVHPSTRRPFGVEPSVSGHTNNFASRSASCLHQSSVREAAYSHLSTTKRQSSSGHAVELYSIPPSSTKSQSQGPVFSCWWLQFRDSEPCSDYCLSHLVNLLQDWGPCTEHGEHHIRIPRTPARVTGGVFLVDKNPHNTTESRLVVDFSQFSRGSARVSWPKFAVPNLQSLTNLLSSNLSWLSLDVSAAFYHIPLHPAAMPHLLVGSSGLSRYVARLSSDSRILDHQYGTLQNLHDSCSRQLYVSLMLLYKTFGRKLHLYSHPIILGFRKIPMGVGLSPFLLAQFTSAICSVVRRAFPHCLAFSYMDDVVLGAKSVQHLESLYTAVTNFLLSLGIHLNPTKTKRWGYSLNFMGYVIGSWGTLPQEHITQKIKQCFRKLPVNRPIDWKVCQRITGLLGFAAPFTQCGYPALMPLYACIQAKQAFTFSPTYKAFLCKQYMNLYPVARQRPGLCQVFADATPTGWGLAIGHQRMRGTFVAPLPIHTAELLAACFARSRSGAKLIGTDNSVVLSRKYTSFPWLLGCAANWILRGTSFVYVPSALNPADDPSRGRLGLCRPLLRLPFLPTTGRTSLYAVSPSVPSHLPDRVHFASPLHVTWKPP

</sequence>

<sequence>

<taxon idref="AY090454_H"/>

MPLSYQHFRRLLLLDN--EAGPLEEELPRLADEDLNHRVAEDLNLQLPNVSIPWTHKVGNFTGLYSSTVPVFNPDWLTPSFPDIHLHQDLIQKCEQFVRPLTKNEVRRLKLIMPARFYPKATKYFPLDKGIKPYYPENVVNHYFKTTHYLHTLWKARILYKRESTHSASFCGSPYSWEQELQHGSTSLNGEKGHGTESLCAQSSGILSRPPVGSTIQSKFQQSRLGLQHKQGQLANGKQGRSGRLWSRVHTPTRWPSGVEPSGTGHSDNLATRSTSRFHQSEVRKETNPSLSTSKGHTSTGHAVELNTVPPSTVGSESQGSVFSCWWLQFRNTEPCSDYCLSHIINLLEDWGPCYEHGEHHIRTPRTPSRVTGGVFLVDKNPHNTTESRLVVDFSQFSRGTTRVSWPKFAVPNLQSLTNLLSSNLSWLSLDVSAAFYHLPLHPAAMPHLLVGSSGLSRYVARVSSTSRIYNHQHGTLQNLHHSCSRNLYVSLLLLYQTFGRKLHLYSHPIILGFRKIPMGVGLSPFLLAQFTSAICSVVRRAFPHCLAFSYMDDLVLGAKSVQHLESLYTAVTNFLLSVGIHLNTAKTKWWGYSLHFMGYIIGSWGTLPQEHIVQKIKNCFRKLPVNRPIDWKVCQRIVGLLGFAAPFTQCGYPALMPLYACITAKQAFVFSPTYKAFLCKQYMNLYPVARQRPGLCQVFADATPTGWGLAIGHQRMRGTFVAPLPIHTAELLAACFARSRSGADIIGTDNSVVLSRKYTSFPWLLGCAANWILRGTSFVYVPSALNPADDPSRGRLGLCRPLLRLPFRPTTGRTSLYADSPPVPFHQPARVHFGSPLHVAWRPP

</sequence>

<sequence>

<taxon idref="AY090457_H"/>

MPLSYQHFRRLLLLDN--EAGPLEEELPRLADEDLNHRVAEDLNLQLPNVSIPWTHKVGNFTGLYSSTVPVFNPDWLTPSFPDIHLHQDLIQKCEQFVGPLTKNEVRRLKLIMPARFYPKVTKYFPLDKGIKPYYPEHVVNHYFKTRHYLHTLWKAGILYKRESTHSASFCGSPYSWEQELQHGSTSLNGEKGHGTESFCAQSSGILSRPPVGSTIQSKFQQSRLGLQHKQGQLANGKQGRSGRLWSRVHTPTRWPSGVEPSGTGHSDNLATRSTSRFHQSEVRKETNPSLSTSKGHTSTGHAVELNTVPPSTVGSESKGSVSSCWWLQFRNTEPCSDYCLSHIINLLEDWGPCYEHGEHHIRTPRTPSRVTGGVFLVDKNPHNTTESRLVVDFSQFSRGTTRVSWPKFAVPNLQSLTNLLSSNLSWLSLDVSAAFYHLPLHPAAMPHLLVGSSGLSRYVARVSSTSRIYNHQHGSLQNLHHSCSRNLYVSLLLLYQTFGRKLHLYSHPIILGFRKIPMGVGLSPFLLAQFTSAICSVVRRAFPHCLAFSYMDDLVLGAKSVQHLESLYTAVTNFLLSVGIHLNTAKTKWWGYSLHFMGYIIGSWGTLPQEHIVQKIKDCFRKLPVNRPIDWKVCQRIVGLLGFAAPFTQCGYPALMPLYACITAKQAFVFSPTYKAFLCKQYMNLYPVARQRPGLCQVFADATPTGWGLAIGHQRMRGTFVAPLPIHTAELLAACFARSRSGADIIGTDNSVVLSRKYTSFPWLLGCAANWILRGTSFVYVPSALNPADDPSRGRLGLCRPLLRLPFRPTTGRTSLYADSPPVPSHLPARVHFASPLHVAWRPP

</sequence>

<sequence>

<taxon idref="AY090460_H"/>

MPLSYQHFRRLLLLDN--EAGPLEEELPRLADEDLNHRVAEDLNLQLPNVSIPWTHKVGNFTGLYSSTIPVFNPDWLTPSFPDIHLHQDLIQKCEQFVGPLTTNERRRLKLIMPARFYPKVTKYFPLDKGIKPYYPENVVNHYFKTRHYLHTLWKAGILYKRESTHSASFCGSPYSWEQELQHGSTSLNGEKGHGTEPFCAQSSGILSRPPVGSTIQSKFQQSRLGLQHKQGQLANGKQGRSGRLRSRVHTPTRWPSGVEPSGTGHSDNLATRSTSCFHQSEVRKKANPSLSTSKGHTSTGHAVELNTVPPSTVGSESKGSVFSCWWLQFRNTEPCSDYCLSHIINLLEDWGPCYEHGEHHIRTPKTPSRVTGGVFLVDKNPHNTTESRLVVDFSQFSRGTTRVSWPKFAVPNLQSLTNLLSSNLSWLSLDVSAAFYHLPLHPAAMPHLLVGSSGLSRYVARVSSTSRIYNHQHGTLQNLHHSCSRNLYVSLLLLYQTFGRKLHLYSHPIILGFRKIPMGVGLSPFLLAQFTSAICSVVRRAFPHCLAFSYMDDLVLGAKSVQHLESLYTAVTNFLLSVGIHLNTAKTKWWGYSLHFMGYIIGSWGTLPQEHIVHKIKDCFRKLPVNRPIDWKVCQRIVGLLGFAAPFTQCGYPALMPLYACITAKQAFVFSPTYKAFLCKQYMNLYPVARQRPGLCQVFADATPTGWGLAIGHQRMRGTFVAPLPIHTAELLAACFARSRSGADIIGTDNSVVLSRKYTSFPWLLGCAANWILRGTSFVYVPSALNPADDPSRGRLGLCRPLLRLPFRPTTGRTSLYADSPPVPSHLPARVHFASPLHVAWRPP

</sequence>

<sequence>

<taxon idref="D00329_B"/>

MPLSYQHFRKLLLLDD--EAGPLEEELPRLADEGLNHRVAEDLNLGNPNVSIPWTHKVGNFTGLYSSTVPVFNPEWQTPSFPDIHLQEDIVDRCKQFVGPLTVNENRRLKLIMPARFYPNVTKYLPLDKGIKPYYPEHVVNHYFQTRHYLHTLWKAGILYKRESTHSASFCGSPYSWEQDLQHGRLVFQTSKRHGDKSFCPQSPGILPRSSVGPCIQSQLRKSRLGPQPTQGQLAGRPQGGSGSIRARIHPSPWGTVGVEPSGSGHTHICASSSSSCLHQSAVRTAAYSPISTSKGHSSSGHAVELHHFPPNSSRSQSQGSVLSCWWLQFRNSKPCSEYCLSHIVNLIEDWGPCAEHGEHRIRTPRTPARVTGGVFLVDKNPHNTTESRLVVDFSQFSRGNTRVSWPKFAVPNLQSLTNLLSSNLSWLSLDVSAAFYHLPLHPAAMPHLLVGSSGLSRYVARLSSNSRIINHQHGTMQDLHNSCSRNLYVSLMLLYKTYGWKLHLYSHPIILGFRKIPMGVGLSPFLLAQFTSAICSVVRRAFPHCLAFSYMDDVVLGAKSVQHLESLYAAVTNFLLSLGIHLNPNKTKRWGYSLNFMGYVIGSWGTWPQDHIVQNFKLCFRKLPVNRPIDWKVCQRIVGLLGFAAPFTQCGYPALMPLYACIQAKQAFTFSPTYKAFLSKQYMTLYPVARQRPGLCQVFADATPTGWGLAIGHQRMRGTFVSPLPIHTAELLAACFARSRSGANLIGTDNSVVLSRKYTSFPWLLGCAANWILRGTSFVYVPSALNPADDPSRGRLGLYRPLLRLPYRPTTGRTSLYADSPSVPSHLPDRVHFASPLHVAWRPP

</sequence>

<sequence>

<taxon idref="X04615_C"/>

MPLSYQHFRKLLLLDD--EAGPLEEELPRLADEGLNRRVAEDLNLGNLNVSIPWTHKVGNFTGLYSSTVPVFNPDWKTPSFPHIHLQEDIINRCQQYVGPLTVNEKRRLKLIMPARFYPNLTKYLPLDKGIKPYYPEYAVNHYFKTRHYLHTLWKAGILYKRETTRSASFCGSPYSWEQELQHGRLVFQTSTRHGDESFCSQSSGILSRSPVGPCVRSQLKQSRLGLQPQQGSLARGKSGRSGSIWSRVHPTTRRPFGVEPSGSGHIDNTASSTSSCLHQSAVRKTAYSHLSTSKRQSSSGHAVELHNIPPSSARSQSEGPIFSCWWLQFRNSKPCSDYCLTHIVNLLEDWGPCTEHGEHNIRIPRTPARVTGGVFLVDKNPHNTTESRLVVDFSQFSRGSTHVSWPKFAVPNLQSLTNLLSSNLSWLSLDVSAAFYHIPLHPAAMPHLLVGSSGLPRYVARLSSTSRNINYQHGTMQNLHDSCSRNLYVSLLLLYKTFGRKLHLYSHPIILGFRKIPMGVGLSPFLLAQFTSAICSVVRRAFPHCLAFSYMDDVVLGAKSVQHLESLFTSITNFLLSLGIHLNPNKTKRWGYSLNFMGYVIGSWGTLPQEHIVQKLKQCFRKLPVNRPIDWKVCQRIVGLLGFAAPFTQCGYPALMPLYACIQSKQAFTFSPTYKAFLCKQYLNLYPVARQRSGLCQVFADATPTGWGLAIGHRRMRGTFVAPLPIHTAELLAACFARSRSGAKLIGTDNSVVLSRKYTSFPWLLGCAANWILRGTSFVYVPSALNPADDPSRGRLGLYRPLLHLPFRPTTGRTSLYAVSPSVPSHLPDRVHFASPLHVAWRPP

</sequence>

<sequence>

<taxon idref="X75657_E"/>

MPLSYQHFRRILLLDE--EAGPLEEELPRLADEDLNRRVAEDLNLQLPNVSIPWTHKVGNFTGLYSSTIPVFNPNWKTPSFPDIHLHQDIINKCEQFVGPLTVNEKRRLNLVMPARFFPISTKYLPLEKGIKPYYPDNVVNHYFQTRHYLHTLWKAGILYKRETTRSASFCGSPYSWEQELHHG-AFLDGPSRMGEEYFHHQSSGIFSRPPVGSSIQSKHQKSRLGPQSQQRPLDRSQQGRSGSIRAWVHSPTRRPFGVEPSGSRHAKNIASRSASCLHQSAVRKAAYPNHSTFERHSSSGHAVEFHNIPPSSAGSQSKRPVFSCWWLQFRNSEPCSDYCLTHLVNLLEDWGPCTEHGKHHIRIPRTPARVTGGVFLVDKNPHNTAESRLVVDFSQFSRGSSRVSWPKFAVPNLQSLTNLLSSNLSWLSLDVSAAFYHLPLHPAAMPHLLVGSSGLSRYVARLSSNSRIINHQYGTLPNLHDSCSRNLYVSLMLLFKTFGRKLHLYSHPIIMGFRKIPMGVGLSPFLLAQFTSAICSVVRRAFPHCLAFSYMDDVVLGAKSVQHLESLYTAVTNFLLSLGIHLNPNKTKRWGYSLNFMGYVIGSWGSLPQEHIIQKIKDCFRKLPVNRPIDWKVCQRIVGLLGFAAPFTQCGYPALMPLYACTQSKQAFTFSPTYKAFLCKQYLNLYPVARQRPGLCQVFADATPTGWGLAIGIQRMRGTFVAPLPIHTAELLAACFARSRSGAKLIGTDNSVVLSRKYTSFPWLLGCAANWILRGTSFVYVPSALNPADDPSRGRLGIYRPLLRLPFQPTTGRTSLYAVSPSVPSHLPDRVHFASPLHVAWRPP

</sequence>

</alignment>

<!-- The unique patterns for all positions -->

<!-- npatterns=307 -->

<patterns id="patterns" from="1">

<alignment idref="alignment"/>

</patterns>

<!-- A prior assumption that the population size has remained constant -->

<!-- throughout the time spanned by the genealogy. -->

<constantSize id="constant" units="substitutions">

<populationSize>

<parameter id="constant.popSize" value="0.1" lower="0.0" upper="Infinity"/>

</populationSize>

</constantSize>

<!-- Generate a random starting tree under the coalescent process -->

<coalescentTree id="startingTree">

<constrainedTaxa>

<taxa idref="taxa"/>

<tmrca monophyletic="false">

<taxa idref="TypeA"/>

</tmrca>

<tmrca monophyletic="false">

<taxa idref="TypeB"/>

</tmrca>

<tmrca monophyletic="false">

<taxa idref="TypeC"/>

</tmrca>

<tmrca monophyletic="false">

<taxa idref="TypeD"/>

</tmrca>

<tmrca monophyletic="false">

<taxa idref="TypeE"/>

</tmrca>

<tmrca monophyletic="false">

<taxa idref="TypeF"/>

</tmrca>

<tmrca monophyletic="false">

<taxa idref="TypeG"/>

</tmrca>

<tmrca monophyletic="false">

<taxa idref="TypeH"/>

</tmrca>

</constrainedTaxa>

<constantSize idref="constant"/>

</coalescentTree>

<treeModel id="treeModel">

<coalescentTree idref="startingTree"/>

<rootHeight>

<parameter id="treeModel.rootHeight"/>

</rootHeight>

<nodeHeights internalNodes="true">

<parameter id="treeModel.internalNodeHeights"/>

</nodeHeights>

<nodeHeights internalNodes="true" rootNode="true">

<parameter id="treeModel.allInternalNodeHeights"/>

</nodeHeights>

</treeModel>

<coalescentLikelihood id="coalescent">

<model>

<constantSize idref="constant"/>

</model>

<populationTree>

<treeModel idref="treeModel"/>

</populationTree>

</coalescentLikelihood>

<!-- The uncorrelated relaxed clock (Drummond, Ho, Phillips & Rambaut, 2006) -->

<discretizedBranchRates id="branchRates">

<treeModel idref="treeModel"/>

<distribution>

<logNormalDistributionModel meanInRealSpace="true">

<mean>

<parameter id="ucld.mean" value="2.0E-5"/>

</mean>

<stdev>

<parameter id="ucld.stdev" value="0.1" lower="0.0" upper="10.0"/>

</stdev>

</logNormalDistributionModel>

</distribution>

<rateCategories>

<parameter id="branchRates.categories" dimension="44"/>

</rateCategories>

</discretizedBranchRates>

<rateStatistic id="meanRate" name="meanRate" mode="mean" internal="true" external="true">

<treeModel idref="treeModel"/>

<discretizedBranchRates idref="branchRates"/>

</rateStatistic>

<rateStatistic id="coefficientOfVariation" name="coefficientOfVariation" mode="coefficientOfVariation" internal="true" external="true">

<treeModel idref="treeModel"/>

<discretizedBranchRates idref="branchRates"/>

</rateStatistic>

<rateCovarianceStatistic id="covariance" name="covariance">

<treeModel idref="treeModel"/>

<discretizedBranchRates idref="branchRates"/>

</rateCovarianceStatistic>

<!-- The JTT substitution model -->

<aminoAcidModel id="aa" type="JTT"/>

<!-- site model -->

<siteModel id="siteModel">

<substitutionModel>

<aminoAcidModel idref="aa"/>

</substitutionModel>

<gammaShape gammaCategories="4">

<parameter id="siteModel.alpha" value="0.5" lower="0.0" upper="100.0"/>

</gammaShape>

<proportionInvariant>

<parameter id="siteModel.pInv" value="0.5" lower="0.0" upper="1.0"/>

</proportionInvariant>

</siteModel>

<treeLikelihood id="treeLikelihood">

<patterns idref="patterns"/>

<treeModel idref="treeModel"/>

<siteModel idref="siteModel"/>

<discretizedBranchRates idref="branchRates"/>

</treeLikelihood>

<tmrcaStatistic id="tmrca(TypeA)">

<mrca>

<taxa idref="TypeA"/>

</mrca>

<treeModel idref="treeModel"/>

</tmrcaStatistic>

<tmrcaStatistic id="tmrca(TypeB)">

<mrca>

<taxa idref="TypeB"/>

</mrca>

<treeModel idref="treeModel"/>

</tmrcaStatistic>

<tmrcaStatistic id="tmrca(TypeC)">

<mrca>

<taxa idref="TypeC"/>

</mrca>

<treeModel idref="treeModel"/>

</tmrcaStatistic>

<tmrcaStatistic id="tmrca(TypeD)">

<mrca>

<taxa idref="TypeD"/>

</mrca>

<treeModel idref="treeModel"/>

</tmrcaStatistic>

<tmrcaStatistic id="tmrca(TypeE)">

<mrca>

<taxa idref="TypeE"/>

</mrca>

<treeModel idref="treeModel"/>

</tmrcaStatistic>

<tmrcaStatistic id="tmrca(TypeF)">

<mrca>

<taxa idref="TypeF"/>

</mrca>

<treeModel idref="treeModel"/>

</tmrcaStatistic>

<tmrcaStatistic id="tmrca(TypeG)">

<mrca>

<taxa idref="TypeG"/>

</mrca>

<treeModel idref="treeModel"/>

</tmrcaStatistic>

<tmrcaStatistic id="tmrca(TypeH)">

<mrca>

<taxa idref="TypeH"/>

</mrca>

<treeModel idref="treeModel"/>

</tmrcaStatistic>

<operators id="operators">

<scaleOperator scaleFactor="0.3812" weight="1">

<parameter idref="siteModel.alpha"/>

</scaleOperator>

<scaleOperator scaleFactor="0.5877" weight="1">

<parameter idref="siteModel.pInv"/>

</scaleOperator>

<scaleOperator scaleFactor="0.75" weight="3">

<parameter idref="ucld.stdev"/>

</scaleOperator>

<swapOperator size="1" weight="10" autoOptimize="false">

<parameter idref="branchRates.categories"/>

</swapOperator>

<randomWalkIntegerOperator windowSize="1.0" weight="10">

<parameter idref="branchRates.categories"/>

</randomWalkIntegerOperator>

<uniformIntegerOperator weight="10">

<parameter idref="branchRates.categories"/>

</uniformIntegerOperator>

<scaleOperator scaleFactor="0.2863" weight="3">

<parameter idref="constant.popSize"/>

</scaleOperator>

<scaleOperator scaleFactor="0.7087" weight="3">

<parameter idref="treeModel.rootHeight"/>

</scaleOperator>

<uniformOperator weight="30">

<parameter idref="treeModel.internalNodeHeights"/>

</uniformOperator>

<subtreeSlide size="695.1087" gaussian="true" weight="15">

<treeModel idref="treeModel"/>

</subtreeSlide>

<narrowExchange weight="15">

<treeModel idref="treeModel"/>

</narrowExchange>

<wideExchange weight="3">

<treeModel idref="treeModel"/>

</wideExchange>

<wilsonBalding weight="3">

<treeModel idref="treeModel"/>

<constantSize idref="constant"/>

</wilsonBalding>

</operators>

<mcmc id="mcmc" chainLength="20000000" autoOptimize="true">

<posterior id="posterior">

<prior id="prior">

<jeffreysPrior>

<parameter idref="constant.popSize"/>

</jeffreysPrior>

<coalescentLikelihood idref="coalescent"/>

</prior>

<likelihood id="likelihood">

<treeLikelihood idref="treeLikelihood"/>

</likelihood>

</posterior>

<operators idref="operators"/>

<log id="screenLog" logEvery="1000">

<column label="Posterior" dp="4" width="12">

<posterior idref="posterior"/>

</column>

<column label="Prior" dp="4" width="12">

<prior idref="prior"/>

</column>

<column label="Likelihood" dp="4" width="12">

<likelihood idref="likelihood"/>

</column>

<column label="Root Height" sf="6" width="12">

<parameter idref="treeModel.rootHeight"/>

</column>

<column label="Rate" sf="6" width="12">

<rateStatistic idref="meanRate"/>

</column>

</log>

<log id="fileLog" logEvery="1000" fileName="HBVncbi.log">

<posterior idref="posterior"/>

<prior idref="prior"/>

<likelihood idref="likelihood"/>

<rateStatistic idref="meanRate"/>

<parameter idref="treeModel.rootHeight"/>

<tmrcaStatistic idref="tmrca(TypeA)"/>

<tmrcaStatistic idref="tmrca(TypeB)"/>

<tmrcaStatistic idref="tmrca(TypeC)"/>

<tmrcaStatistic idref="tmrca(TypeD)"/>

<tmrcaStatistic idref="tmrca(TypeE)"/>

<tmrcaStatistic idref="tmrca(TypeF)"/>

<tmrcaStatistic idref="tmrca(TypeG)"/>

<tmrcaStatistic idref="tmrca(TypeH)"/>

<parameter idref="constant.popSize"/>

<parameter idref="siteModel.alpha"/>

<parameter idref="siteModel.pInv"/>

<parameter idref="ucld.mean"/>

<parameter idref="ucld.stdev"/>

<rateStatistic idref="coefficientOfVariation"/>

<rateCovarianceStatistic idref="covariance"/>

<treeLikelihood idref="treeLikelihood"/>

<coalescentLikelihood idref="coalescent"/>

</log>

<logTree id="treeFileLog" logEvery="1000" nexusFormat="true" fileName="HBVncbi.trees" sortTranslationTable="true">

<treeModel idref="treeModel"/>

<discretizedBranchRates idref="branchRates"/>

<posterior idref="posterior"/>

</logTree>

</mcmc>

<report>

<property name="timer">

<object idref="mcmc"/>

</property>

</report>

</beast>
