# Supplementary figures and images for: Protein X of Hepatitis B Virus: Origin and Structure Similarity with the Central Domain of DNA Glycosylase
Source: PLoS One. 2011 Aug 5;6(8):e23392. doi: 10.1371/journal.pone.0023392 (PMC3153941; doi:10.1371/journal.pone.0023392)

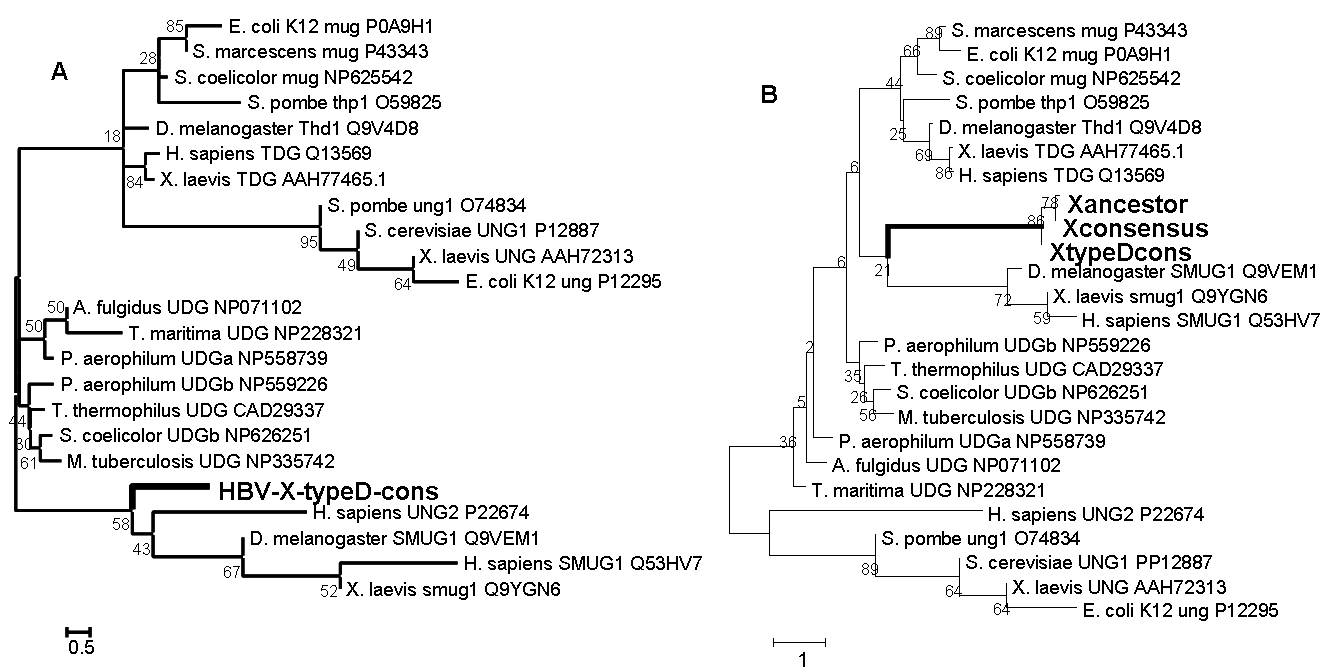

Supplement: File S5 — Evolutionary position of HBx among DNA glycosylases. A: A phylogenetic tree of MUG proteins was constructed according to Cortazar et al. (2007, DNA Repair, 6, 489–504) with HBx (D-type consensus sequence) indicated in bold typeface. B: A similar tree (the same set of sequences with ancestor and consensus HBx derived from the NCBI reference set of HBV) was constructed after re-alignment by ProbCons (Do et al., 2005, Genome Res., 156, 2, 330–340) followed by rounds of manual refinement with special attention at gap borders. (TIF) [file pone.0023392.s005.tif]
